# Benzimidazole-Based N,O Boron Complexes as Deep Blue Solid-State Fluorophores

**DOI:** 10.3390/ma14154298

**Published:** 2021-07-31

**Authors:** Patrícia A. A. M. Vaz, João Rocha, Artur M. S. Silva, Samuel Guieu

**Affiliations:** 1 LAQV-REQUIMTE, Department of Chemistry, University of Aveiro, 3810-193 Aveiro, Portugal; patriciavaz@ua.pt (P.A.A.M.V.); artur.silva@ua.pt (A.M.S.S.); 2 CICECO, Aveiro Institute of Materials, Department of Chemistry, University of Aveiro, 3810-193 Aveiro, Portugal; rocha@ua.pt

**Keywords:** fluorescence, benzimidazole, boranil

## Abstract

Benzimidazole-based boranils were designed and synthesized in order to assess the influence of halogen substituents on their optoelectronic properties. All compounds are photoluminescent in solution and solid state. Compared to the free ligands, the new boranils emit at a lower wavelength, by elimination of the excited-state intramolecular proton transfer observed with the ligands. In the solid state, some of the boranils exhibit a deep blue emission, presenting Commission Internationale de l’Éclairage (CIE) coordinates with an x-component of less than 0.16 and a y-component smaller than 0.04, highly desired values for the development of blue emitting materials.

## 1. Introduction

Benzimidazole and other azole derivatives raise much interest due to their luminescence properties, and as promising candidates for the development of sensors and solar cells [1,2,3,4,5]. They are receiving increasing attention due to their non-linear optical properties [6,7] and excellent thermal stability [8,9]. Widely reported in the literature, these compounds are also of interest because of their intense emission via excited-state intramolecular proton transfer (ESIPT) [10,11,12]. Due to their optical properties, fully conjugated core, and possibility of functionalization of the aromatic ring, benzimidazole derivatives are privileged ligands to develop materials with bright blue emissions [7,13]. Another advantage of these compounds is that they have an excellent coordination ability with a wide range of transition metals, and may present interesting selectivity and sensitivity toward different metal cations [14].

**Figure 1:**
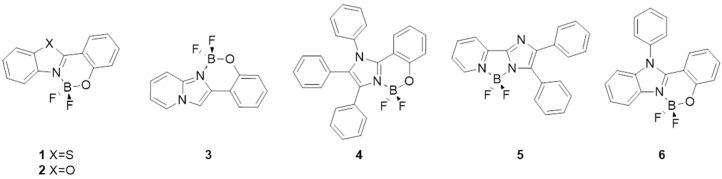
Azole-based organoboron compounds suitable for optoelectronic applications.

Complexation with boron is widely used in the synthesis of luminescent dyes. Boron is a nontoxic alternative to transition metals, and boron complexes have been studied for their excellent photoluminescent properties [15,16,17,18,19]. The latter are cheaper than certain transition metals, and because of the strong covalent bonds with the ligand, they may allow the synthesis of more stable luminescent materials [20,21,22]. For example, difluoroboron complexation enabled thermally activated delayed fluorescence, which find interesting applications in organic light-emitting diodes [23]. Benzimidazoles bearing an *ortho*-hydroxy aromatic substituent are interesting fluorescent molecules (Figure 1) because of their large Stokes shifts due to their ESIPT [24,25]. This mechanism can be inhibited by binding a boron atom to the hydroxy group and the nitrogen atom of the azole unit: longer emission wavelengths are lost, resulting in a hypsochromic shift [26]. Derivatives of 2-(2-hydroxyphenyl)benzothiazole **1** and 2-(2-hydroxyphenyl)benzoxazole **2** are examples of compounds explored for blue emission [27,28,29]. Recently, boron complexes **3** incorporating imidazo[1,5-*a*]pyridine as a *N,O*-type ligand were reported [30], as well as tetraaryl substituted imidazole boron difluoride complexes **4** [31]. Examples of reported boron complexes based on bidentate 2-(2-pyridyl)imidazole **5**, and *N*-alkylated 2-(2-hydroxyphenyl)benzimidazole **6**, showed emissions in both solution and solid state [32,33].

Although imidazole-phenol complexes have been studied for application in electronic materials [34], the nitrogen atom not involved in the complexation usually bears a substituent, and if 2-(1*H*-benzo[*d*]imidazol-2-yl)phenol has been studied for boron complexation [35], to the best of our knowledge no studies of their photophysical properties have been performed.

The introduction of halogen atoms on the backbone of fluorophores is a strategy used to promote their phosphorescence. Indeed, the heavy atom effect can promote intersystem crossing between the excited singlet and triplet states. If a halogen bond is formed in the solid state, the effect can be even more pronounced, allowing the observation of phosphorescence in purely organic solids at room temperature [36]. This may find applications in the development of organic light emitting devices.

**Figure 2:**
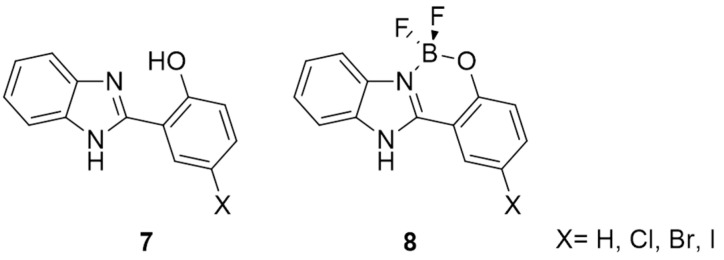
Halogenated boron complexes **8** based on 2-(1H-benzo[d]imidazol-2-yl)phenol **7** studied in this work.

Here, we wish to report the synthesis and characterization of boron complexes **8** based on halogenated 2-(1*H*-benzo[*d*]imidazol-2-yl)phenol **7** (Figure 2), and the study of their optoelectronic properties. In the design of the fluorophores, the nitrogen not involved in the complexation was kept unsubstituted to minimize the conjugation of the backbone, in order to produce a blue emission. Halogen atoms have been introduced on one aromatic ring, in an attempt to promote intersystem crossing and therefore phosphorescent dyes.

## 2. Materials and Methods

### 2.1. Materials and Methods

All reagents were purchased from Sigma-Aldrich (St. Louis, MO, USA) and used without any further purification. The final compounds were purified by crystallization. ^1^H, ^19^F and ^13^C NMR spectra were recorded on Bruker 300 or 500 [300.13 MHz (^1^H), 282.41 (^19^F), 75.47 MHz (^13^C) or 500.13 MHz (^1^H)]. Unequivocal ^13^C assignments were made on the basis of 2D Heteronuclear Single Quantum Coherence (HSQC) (^1^H/^13^C) and Heteronuclear Multiple Bond Correlation (HMBC) experiments. DMSO-*d6* was used as the solvent and tetramethylsilane (TMS) as the internal standard. Chemical shifts δ are reported in parts per million (ppm) relative to TMS (δ = 0), and the values of coupling constants (*J*) are given in Hertz (Hz). High-resolution mass spectra (HRMS-ESI^+^) were recorded on an LTQ Orbitrap™ XL hybrid mass spectrometer (Thermo Fischer Scientific, Bremen, Germany) controlled by LTQ Tune Plus 2.5.5 and Xcalibur 2.1.0. The capillary voltage of the electrospray ionization source (ESI) was set to 3.1 kV. Melting points were determined on a BUCHI Melting point apparatus (BÜCHI Labortechnik AG, Flawil, Switzerland) and are uncorrected. The ultraviolet-visible (UV-Vis) spectra in dimethyl sulfoxide solutions were obtained on a Shimadzu UV-2501 PC spectrophotometer (1 cm path length quartz cell, Shimatzu, Nakagyō-ku, Japan) and the UV-Vis absorption spectra of the solids were measured at room temperature on a JASCO V-560 instrument (JASCO Inc., Easton, MD, USA). The excitation and emission spectra, also in dimethyl sulfoxide solutions, were recorded on a Jobin Yvon FluoroMax-3 spectrofluorometer (Horiba, Lier, Belgium) and a JASCO spectrofluorometer (JASCO Inc., Easton, MD, USA). Fluorescence quantum yields φF were determined using fluorescein in 0.1 M NaOH water solution as a fluorescence standard. The absolute emission quantum yields in the solid state were measured at room temperature using a system (Quantaurus-QY Plus C13534, Hamamatsu, Shizuoka, Japan) with a 150 W xenon lamp coupled to a monochromator for wavelength discrimination, an integrating sphere as the sample chamber, and a multichannel analyzer for signal detection. The method is accurate to within 10%.

### 2.2. Synthesis

#### 2.2.1. General Procedure for the Synthesis of Ligands **7a**–**d**

The general procedure is described here for **7a**: salicylaldehyde (1.0 equiv., 1.0 mL, 9.6 mmol) and *o*-phenylenediamine (1.5 equiv., 1.6 g, 14.4 mmol) were mixed in methanol (2 mL). After everything dissolved, a red solid immediately formed. Acetic acid (20 mL) was added and the reaction mixture was stirred at room temperature for 4 h. The reaction was quenched by adding water (60 mL), the solid was collected by filtration and washed with methanol (3 × 20 mL). The solid obtained was purified by flash column chromatography over silica gel, eluent hexane/ethyl acetate (2:1 *v*/*v*), to afford the product **7a** as off-white crystals after crystallization in chloroform.

2-(1*H*-benzo[*d*]imidazol-2-yl)phenol **7a** [37]

The compound **7a** was obtained as colorless microcrystals (1.25 g, 5.94 mmol, 62%). M.P.: 242–243 °C; ^1^H NMR (300 MHz, DMSO-*d6*) δ 13.17 (br s, 2H, OH and NH), 8.05 (dd, ^3^*J*_H-H_ 7.8, ^4^*J*_H-H_ 1.4 Hz, 1H, Harom), 7.66 (br s, 2H, Harom), 7.38 (ddd, ^3^*J*_H-H_ 8.6, ^3^*J*_H-H_ 7.1, ^4^*J*_H-H_ 1.6 Hz, 1H, Harom), 7.30–7.27 (m, 2H, Harom), 7.05–6.99 (m, 2H, Harom). HRMS-ESI^+^ m/z for [C_13_H_10_N_2_O + H]^+^ calcd 211.0866, found 221.0868.

2-(1*H*-benzo[*d*]imidazol-2-yl)-4-chlorophenol **7b** [37]

The compound **7b** was obtained as colorless microcrystals (90 mg, 0.36 mmol, 60%). M.P.: 306–307 °C; ^1^H NMR (500 MHz, DMSO-*d6*) δ 13.27 (br s, 2H, OH and NH), 8.17 (d, ^4^*J*_H-H_ 2.6 Hz, 1H, Harom), 7.68 (br s, 2H, Harom), 7.42 (dd, ^3^*J*_H-H_ 8.8, ^4^*J*_H-H_ 2.6 Hz, 1H, Harom), 7.31 (br s, 2H, Harom), 7.08 (d, ^3^*J*_H-H_ 8.8, 1H, Harom). HRMS-ESI^+^ m/z for [C_13_H_9_ClN_2_O + H]^+^ calcd 245.0476, found 245.0479.

2-(1*H*-benzo[*d*]imidazol-2-yl)-4-bromophenol **7c**

The compound **7c** was obtained as a brown solid (90 mg, 0.32 mmol, 63%). M.P.: 311–312 °C; ^1^H NMR (300 MHz, DMSO-*d6*) δ 13.30 (br s, 2H, OH and NH), 8.29 (d, ^4^*J*_H-H_ 2.5 Hz, 1H, Harom), 7.68 (dd, ^3^*J*_H-H_ 5.9 Hz, ^4^*J*_H-H_ 3.2 Hz, 2H, Harom), 7.52 (dd, ^3^*J*_H-H_ 8.8, ^4^*J*_H-H_ 2.5 Hz, 1H, Harom), 7.30 (dd, ^3^*J*_H-H_ 5.9 Hz, ^4^*J*_H-H_ 3.2 Hz, 2H, Harom), 7.02 (d, ^3^*J*_H-H_ 8.8, 1H, Harom). ^13^C NMR (75 MHz, DMSO-*d6*) δ 157.2 (1C, C-O), 150.3 (1C, C = N), 134.0 (1C, CHarom), 128.5 (1C, CHarom), 123.2 (4C, CHarom), 119.6 (1C, CHarom), 114.7 (1C, C-Br), 112.4 (2C, Carom) 110.2 (1C, Carom). HRMS-ESI^+^ m/z for [C_13_H_9_BrN_2_O + H]^+^ calcd 288.9971, found 288.9975.

2-(1*H*-benzo[*d*]imidazol-2-yl)-4-iodophenol **7d**

The compound **7d** was obtained as a brown solid (95 mg, 0.2 mmol, 68%). M.P.: 254–255 °C; ^1^H NMR (300 MHz, DMSO-*d6*) δ 13.28 (br s, 2H, OH and NH), 8.42 (d, ^4^*J*_H-H_ 2.2 Hz, 1H, Harom), 7.67 (br s, 2H, Harom), 7.65 (dd, ^3^*J*_H-H_ 8.7, ^4^*J*_H-H_ 2.2 Hz, 1H, Harom), 7.30 (dd, ^3^*J*_H-H_ 6.1 Hz, ^4^*J*_H-H_ 2.4 Hz, 2H, Harom), 6.89 (d, ^3^*J*_H-H_ 8.7 Hz, 1H, Harom). ^13^C NMR (75 MHz, DMSO -*d6*) δ 157.7 (1C, C-O), 150.2 (1C, C = N), 139.7 (1C, CHarom), 134.2 (1C, CHarom), 123.1 (4C, CHarom), 119.9 (1C, CHarom), 115.3 (1C, C-I), 110.4 (2C, Carom), 80.9 (1C, Carom). HRMS-ESI^+^ m/z for [C_13_H_9_IN_2_O + H]^+^ calcd 336.9832, found 336.9833.

#### 2.2.2. General Procedure for the Synthesis of Complexes **8a–d**

The typical synthetic procedure of benzimidazole-based *N*,*O*-chelated boron complexes is described as following for **8a**: BF_3_·OEt_2_ (3 equiv., 0.06 mL, 0.6 mmol) was added dropwise at room temperature to a stirred mixture of ligand **7a** (0.2 mmol) in anhydrous tetrahydrofuran (2 mL), and the reaction mixture was stirred overnight. The solvent was removed under reduced pressure and the solid obtained was washed with anhydrous diethyl ether several times to obtain the complex.

6,6-difluoro-6,12-dihydrobenzo[*e*]benzo[4,5]imidazo[1,2-*c*][1,3,2]oxazaborinin-7-ium-6-uide **8a** [35]

The compound **8a** was obtained as a white solid (62 mg, 0.2 mmol, 100%). M.P.: 348–350 °C. ^1^H NMR (300 MHz, DMSO-*d6*) δ 14.65 (b s, 1H, NH), 8.05 (dd, ^3^*J*_H-H_ 8.1 Hz, ^4^*J*_H-H_ 1.5 Hz, 1H, Harom), 7.85 (dd, ^3^*J*_H-H_ 6.2 Hz, ^4^*J*_H-H_ 2.4 Hz, 2H, Harom), 7.59 (ddd, ^3^*J*_H-H_ 7.8 Hz, ^3^*J*_H-H_ 6.4 Hz, ^4^*J*_H-H_ 1.3 Hz, 1H, Harom), 7.55 (dd, ^3^*J*_H-H_ 6.2 Hz, ^4^*J*_H-H_ 2.4 Hz, 2H, Harom), 7.21 (dd, ^3^*J*_H-H_ 9.0 Hz, ^4^*J*_H-H_ 0.7 Hz, 1H, Harom), 7.15 (ddd, ^3^*J*_H-H_ 7.5 Hz, ^3^*J*_H-H_ 6.2 Hz, ^4^*J*_H-H_ 0.9 Hz, 1H, Harom). ^13^C NMR (75 MHz, DMSO *d-6*) δ 157.1 (1C, C-O), 146.8 (1C, C = N), 135.2 (1C, CHarom), 131.2 (2C, Carom), 129.3 (1C, CHarom), 125.9 (2C, CHarom), 120.0 (1C, CHarom), 117.3 (1C, CHarom), 114.1 (2C, CHarom), 109.2 (1C, Carom).^19^F NMR (282 MHz, DMSO-*d6*) δ -132.2 (dd, *J* 27.7 Hz, *J* 10.7 Hz, 2F). HRMS-ESI+ m/z for [C_13_H_9_BF_2_N_2_O + H] ^+^ calcd 259.0854, found 259.0853.

2-Chloro-6,6-difluoro-6,12-dihydrobenzo[*e*]benzo[4,5]imidazo[1,2-*c*][1,3,2]oxazaborinin-7-ium-6-uide **8b**

The compound **8b** was obtained as a white solid (29 mg, 0.099 mmol, 46%), M.P.: 308–309 °C. ^1^H NMR (300 MHz, DMSO-*d6*) δ 14.71 (s, 1H, NH), 8.21 (d, ^4^*J*_H-H_ 2.6 Hz, 1H, Harom), 7.85–7.78 (m, 2H, Harom), 7.61 (dd, ^3^*J*_H-H_ 8.9 Hz, ^4^*J*_H-H_ 2.6 Hz, 1H, Harom), 7.49–7.57 (m, 2H, Harom), 7.16 (d, 3*J*_H-H_ 8.9 Hz, 1H, Harom). ^13^C NMR (75 MHz, DMSO *d-6*) δ 155.3 (1C, *C*-O), 146.1 (1C, C = N), 134.5 (1C, *C*Harom), 132.2 (2C, Carom), 125.8 (1C, *C*Harom), 125.2 (1C, *C*Harom), 124.9 (1C, *C*Harom), 123.3 (1C, *C*-Cl), 121.2 (1C, *C*Harom), 115.1 (1C, *C*Harom), 113.4 (1C, *C*Harom), 110.0 (1C, Carom). ^19^F NMR (282 MHz, DMSO-*d6*) δ -132.1 (dd, *J* 25.4 Hz, *J* 8.7 Hz, 2F). HRMS-ESI+ m/z for [C_13_H_8_B^37^ClF_2_N_2_O + H] ^+^ calcd 293,0465, found 293,0466.

2-Bromo-6,6-difluoro-6,12-dihydrobenzo[*e*]benzo[4,5]imidazo[1,2-*c*][1,3,2]oxazaborinin-7-ium-6-uide **8c**

The compound **8c** was obtained as a white solid (34 mg, 0.10 mmol, 81%). M.P.: 395–396 °C. ^1^H NMR (300 MHz, DMSO-*d6*) δ 14.75 (s, 1H, NH), 8.34 (d, ^4^*J*_H-H_ 2.5 Hz, 1H, Harom), 7.78–784 (m, 2H, Harom), 7.72 (dd, ^3^*J*_H-H_ 8.9 Hz, ^4^*J*_H-H_ 2.5 Hz, 1H, Harom), 7.49–7.58 (m, 2H, Harom), 7.10 (d, ^3^*J*_H-H_ 8.9 Hz, 1H, Harom). ^13^C NMR (75 MHz, DMSO *d-6*) δ 155.6 (1C, *C*-O), 146.0 (1C, C = N), 137.2 (1C, *C*Harom), 132.3 (2C, Carom), 127.8 (1C, *C*Harom), 125.8 (1C, *C*Harom), 125.2 (1C, *C*harom), 123.3 (1C, *C*-Br), 121.6 (1C, *C*Harom), 115.1 (1C, *C*Harom), 113.4 (1C, *C*Harom), 110.7 (1C, Carom). ^19^F NMR (282 MHz, DMSO-*d6*) δ -132.1 (dd, *J* 25.4 Hz, *J* 8.7 Hz, 2F). MS-ESI+ m/z for [C_13_H_8_BBrFN_2_O] ^+^ 317.0 (^79^Br), 319.0 (^81^Br). HRMS-ESI- m/z for [C_13_H_8_BBrF_2_N_2_O-H]-calcd 334.9808, found 334.9827.

6,6-difluoro-2-iodo-6,12-dihydrobenzo[*e*]benzo[4,5]imidazo[1,2-*c*][1,3,2]oxazaborinin-7-ium-6-uide **8d**

The compound **8d** was obtained as a brown solid (0.200 g, 0.5 mmol, 79%). M.P.: 379–380 °C. ^1^H NMR (300 MHz, DMSO-*d6*) δ 14.68 (s, 1H, NH), 8.48 (d, ^4^*J*_H-H_ 2.2 Hz, 1H, Harom), 7.86 (dd, ^3^*J*_H-H_ 8.7 Hz, ^4^*J*_H-H_ 2.2 Hz, 1H, Harom), 7.78–7.81 (m, 2H, Harom), 7.49–7.58 (m, 2H, Harom), 6.97 (d, ^3^*J*_H-H_ 8.7, 1H, Harom). ^13^C NMR (75 MHz, DMSO-*d6*) δ 156.1 (1C, C-O), 142.8 (1C, C = N), 142.8 (1C, CHarom), 133.6 (1C, CHarom), 123.2 (1C, Carom), 132.0 (1C, Carom), 121.7 (1C, CHarom), 125.8 (1C, CHarom), 125.2 (1C, CHarom), 115.1 (1C, CHarom), 113.3 (1C, CHarom), 108.2 (1C, Carom), 81.6 (1C, C-I). ^19^F NMR (282 MHz, DMSO-*d6*) δ -132.0 (dd, *J* 25.4 Hz, *J* 8.7 Hz, 2F). HRMS-ESI^+^ m/z for [C_13_H_8_BF_2_IN_2_O + Na]^+^, calcd 406,9640, found 406,9639.

## 3. Results and Discussion

### 3.1. Synthesis

**Scheme 1:**
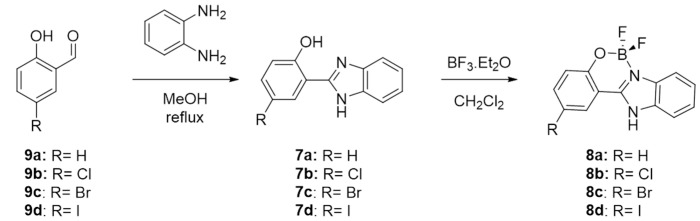
Synthetic procedure for the synthesis of fluoroborates complexes **8a**–**d**.

The synthetic procedure for the synthesis of fluoroborates complexes **8a**–**d** is described in Scheme 1.

Because benzimidazole rings are important building blocks for therapeutic drugs and optoelectronic materials, several strategies for their synthesis are reported in the literature [38]. The most common procedures call for the cyclocondensation of *o*-phenylenediamine derivatives with carboxylic acids under strongly acidic conditions, or with aldehydes under oxidative conditions. Although several oxidative and catalytic reagents have been employed in the synthesis of benzimidazoles from aldehydes [39], some of these methods have certain disadvantages, such as requiring large quantities of reagent, the high cost of the catalysts, prolonged reaction times, occurrence of side reactions, the harsh reaction conditions used, strong oxidizing nature of the reagents, or the use of toxic metal salts. For the synthesis of ligands **7a**–**d** we have chosen the classical cyclocondensation of *o*-phenylenediamine with the corresponding salicylaldehydes **9a**–**d** under oxidative conditions, using the atmospheric oxygen as the oxidant, and promoting the cyclization reaction by adding acetic acid. The reaction starts with the formation of a red solid (the intermediate Schiff base), which disappears after the addition of acetic acid. The reaction solution then became strongly photoluminescent, indicating the formation of the benzimidazole ring.

The synthesis of the fluoroborates complexes **8b**–**d** followed the conditions described for **8a**. Because this compound was obtained with a good yield, the conditions were maintained for the other complexes. Briefly, the ligands **7a**–**d** were dissolved in THF and three equivalents of boron trifluoride diethyl etherate were added at room temperature. The complexes were obtained as off-white solids after evaporation of the reaction mixture under reduced pressure and after several wash cycles with diethyl ether. The instability of the complexes, which are sensitive to hydrolysis, made their isolation difficult, and their purification should be done as quickly as possible. Attempts to purify the complexes using silica gel column chromatography were unsuccessful, and a mixture of the complex and the ligand was systematically recovered. Additionally, the hydrolysis of the complexes was also observed during NMR characterization, if the spectrum was not recorded just after the preparation of the solution. All compounds were fully characterized (see Supplementary Materials).

### 3.2. Photophysical Properties

**Figure 3:**
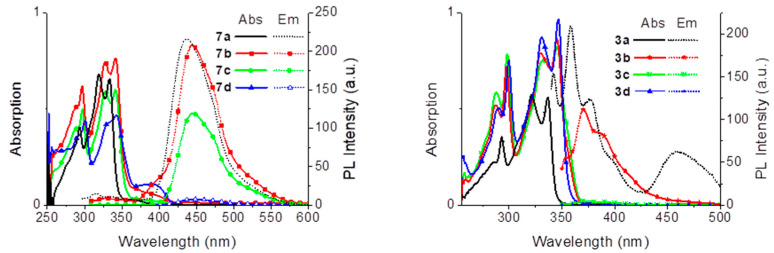
Absorption (full lines) and emission spectra (dotted lines) of compounds **7a**–**d** and compounds **8a**–**d**, in DMSO (at 1 × 10^−5^ mol L^−1^).

The absorption and emission spectra of benzimidazoles **7a**–**d** were recorded in anhydrous DMSO solution and are shown in Figure 3. DMSO was selected to maximize the solubility of both the free ligands and the boron complexes. The solutions were freshly prepared and used immediately, to prevent the hydrolysis of the complexes. The photophysical properties of compounds **7a**–**d** are summarized in Table 1.

The ligand **7a** exhibits a maximum absorption peak at 333 nm, which is ascribed to the π → π * transition (Figure 3). For the halogenated derivatives **7b**–**d**, the maximum absorption wavelength is slightly red shifted, by ca. 10 nm. This small shift can be related to the presence of electron withdrawing groups (–Cl, –Br and I) that decreases the Highest Occupied Molecular Orbital-Lowest Unoccupied Molecular Orbital (HOMO-LUMO) energy gap of substituted derivatives [40,41]. In these kind of compounds, ESIPT is strongly influenced by the nature and position of the substituents, which adjust the strength of the hydrogen bond [42,43,44].

All compounds are fluorescent in DMSO solution and show large Stokes’ shifts of ca. 125 nm, characteristic of fluorophores presenting ESIPT [43,45,46]. The maximum emission wavelength of **7b**–**d** is also slightly red shifted (5–10 nm) moving down the halogen series (Figure 3). The quantum yields are, however, very different, and for **7a** and **7b** they are very good, 93% and 95%, respectively. The quenching observed for **7c** (quantum yield 33%) and **7d** (quantum yield 4%) results from the expected internal heavy atom effect [47], which is stronger with the iodine than with the bromine substituents. All ligands **7a**–**d** exhibit a bright blue fluorescence under irradiation at 254 or 365 nm (Table 2)**.**

The absorption and emission spectra of boron complexes **8a**–**d** in anhydrous DMSO are displayed in Figure 3. Their maximum absorption energy is also ascribed to the π → π * transition [48]. The emission and absorption spectra of the complexes are mirror images and display a small Stokes shift between 16 and 26 nm, indicating the absence of major internal conversions.

**Figure 4:**
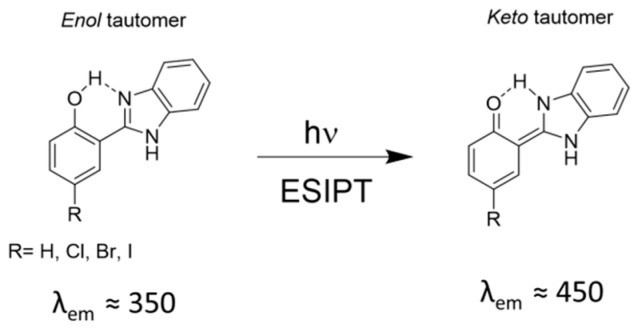
ESIPT fluorescence of benzimidazol derivatives.

The ESIPT (Figure 4) witnesses the transfer of the proton from the hydroxy to the imidazole-*N* moiety through a pre-existing hydrogen bond, and is responsible for the large Stokes’ shift observed for the ligands. The complexation of boron supresses this phenomenon, as the hydrogen atom is no longer present and, as a consequence, the emission profile is dramatically altered. Here, the halogen atoms have a more significant effect on the emission properties: the quantum yields of **8b**–**d** decrease significantly relatively to **8a**. The small Stokes’ shifts and the spectral overlap may lead to the self-quenching of fluorescence for compounds **8a**–**d,** and the halogen atoms may promote the intersystem crossing, leading to a quenching of the fluorescence instead of the expected promotion of phosphorescence.

**Figure 5:**
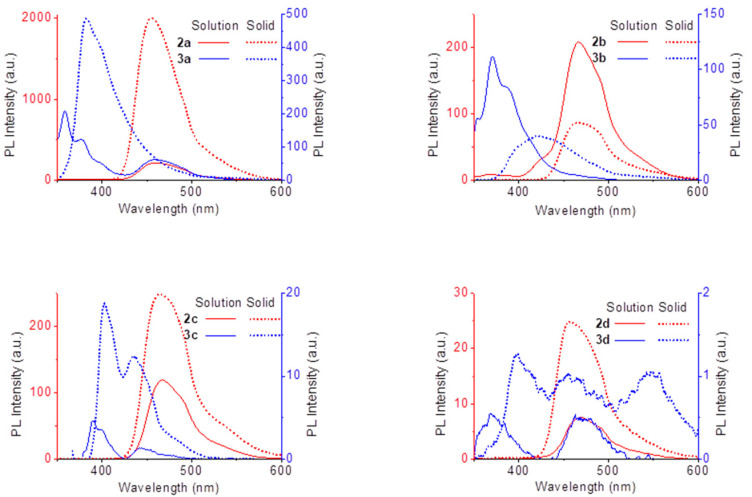
Emission spectra of ligands (red) and complexes (blue), in DMSO solutions (1.0 × 10^−5^ M, full line) and solid state (dotted line).

Figure 5 depicts the emission spectra of complexes and ligands in the solid state and in DMSO solution. The emissions of the complexes are mirror images of their absorption, with structured emission bands, which is not the case for the ligands. This has been observed before for similar fluorophores [32]. While the solid state and solution emission spectra of the ligands present the same profile, the complexes do not. The emission maxima of the latter are red shifted in the solid state relatively to solution. This indicates possible intermolecular interactions in the solid state, such as π···π stacking, which may be responsible for both the red shift and the quenching observed.

**Figure 6:**
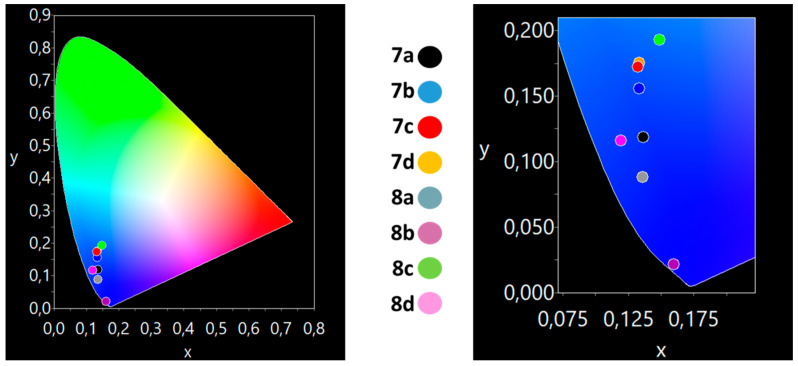
The CIE coordinates for the compounds **7a**–**d** and **8a**–**d** in DMSO solution, showing the deep-blue color of **8b**.

**Figure 7:**
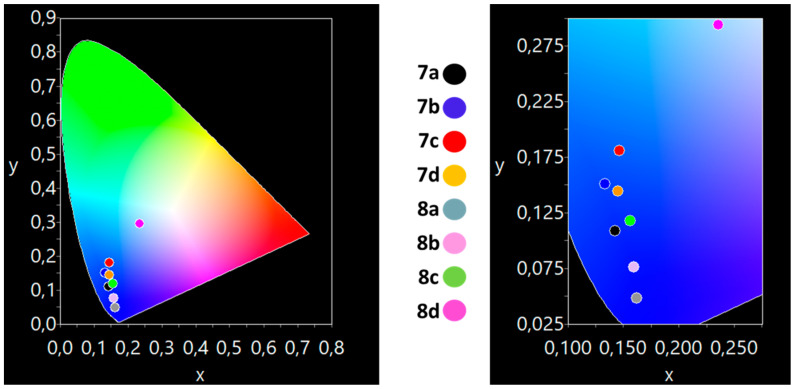
The CIE coordinates for the compounds **7a**–**d** and **8a**–**d** in solid state, showing the deep-blue color of **8a**–**b**.

All compounds exhibit a blue emission in solution and solid state (Figure 6 and Figure 7). In solution, **8b**, with a moderate quantum yield of 26%, exhibits a deep blue emission with CIE coordinates of (0.16, 0.02) with a y-component smaller than 0.04, that is highly desirable and follows the specifications of the European Broadcast Union (EBU) television (0.15, 0.06) [49,50]. In the solid state, all complexes emit a blue color under a UV lamp (Table 2). Both **8a** (0.16, 0.04) and **8b** (0.16, 0.08) present a deep blue color in the solid state.

## 4. Conclusions

Boron complexes based on halogenated 2-(1*H*-benzo[*d*]imidazol-2-yl)phenol were synthesized and characterized. All the halogenated benzimidazole complexes exhibit a blue emission in both DMSO solution and solid state, with moderate quantum yields, demonstrating that keeping the conjugation minimal is an adequate strategy to tune the emission color. On the other hand, the introduction of halogen substituents lowered their quantum yields, and did not promote their phosphorescence in the solid state, making these compounds less promising for electroluminescence. Unfortunately, the instability of the complexes, which are sensitive to hydrolysis, may prevent their applications as emissive materials. Nevertheless, some of them present a deep blue emission, making them interesting starting points for the development of emissive materials.

## Figures and Tables

**Table 1:** Optical properties of compounds **7a**–**d** and **8a**–**d**.

Dye	λ_abs_ (nm) ^1^	ε (M^−1^ cm^−1^) ^2^	λ_em_ (nm) ^3^	δλ_st_	φ_f_^4^ (%)	Solvent
**7a**	333	65,600	460	127	93	DMSO
	325	-	455	130	39	Solid
**7b**	341	76,500	466	125	95	DMSO
	325	-	466	141	18	Solid
**7c**	341	55,700	467	126	33	DMSO
	325	-	466	141	5	Solid
**7d**	343	46,800	470	127	4	DMSO
	325	-	500	175	<1	Solid
**8a**	336	55,300	360	24	75	DMSO
	325	-	380	55	12	Solid
**8b**	344	83,900	370	26	26	DMSO
	325	-	420	95	2	Solid
**8c**	345	82,300	361	16	1	DMSO
	325	-	395	70	2	Solid
**8d**	345	91,300	370	25	<1	DMSO
	325	-	500	175	<1	Solid

^1^ Absorption maximum. ^2^ Molar absorption coefficients. ^3^ Fluorescence maximum. ^4^ In solution: determined by comparison with fluorescein (φ_f_ = 0.90 in water with NaOH 0.1 mol L^−1^) [39] at room temperature; in the solid state: measured using an integrating sphere, the excitation wavelength was 375 nm.

**Table 2:** Photographs of the ligands **7a**–**d** and fluoroborates complexes **8a**–**d** in solid state under UV lamp.

Compound	Day Light	254 nm	365 nm
**7a**	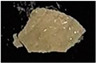	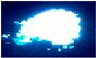	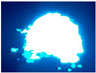
**7b**	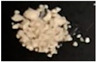	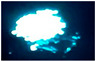	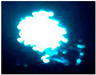
**7c**	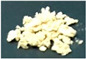	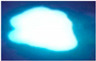	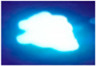
**7d**	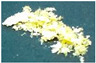	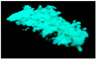	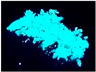
**8a**	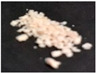	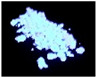	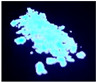
**8b**	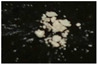	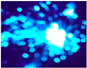	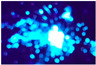
**8c**	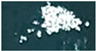	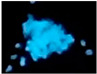	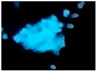
**8d**	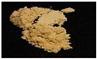	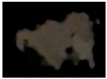	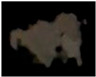

## Data Availability

The data presented in this study are available on request from the corresponding author.

## Supplementary Materials

The following are available online at https://www.mdpi.com/article/10.3390/ma14154298/s1: NMR spectra of all new compounds; absorption, emission and excitation spectra in solution; excitation and emission spectra in solid state.

## References

[1] Cansu-ergun E.G. (2018). Chemical insight into benzimidazole containing donor-acceptor-donor type π-conjugated polymers: Benzimidazole as an acceptor. Polym. Rev..

[2] Burganov T.I., Zhukova N.A., Mamedov V.A., Bannwarth C., Grimme S., Katsyuba S.A. (2017). Benzimidazolylquinoxalines: Novel fluorophores with tuneable sensitivity to solvent effects. Phys. Chem. Chem. Phys..

[3] Xu B., Li Y., Song P., Ma F., Sun M. (2017). Photoactive layer based on T-shaped benzimidazole dyes used for solar cell: From photoelectric properties to molecular design. Nat. Sci. Rep..

[4] Reddy S.S., Cho W., Sree V.G., Jin S. (2016). Multi-functional highly efficient bipolar 9,9-dimethyl-9,10-dihydroacridine/imidazole-based materials for solution-processed organic light-emitting diode applications. Dyes Pigment..

[5] Bodedla G.B., Thomas K.R.J., Fan M., Ho K. (2016). Benzimidazole-Branched Isomeric Dyes: Effect of Molecular Constitution on Photophysical, Electrochemical, and Photovoltaic Properties. J. Org. Chem..

[6] Kulhánek J., Bures F., Pytela O., Mikysek T., Ludvík J. (2011). Imidazole as a donor/acceptor unit in charge-transfer chromophores with extended π-linkers. Chem. Asian J..

[7] Lai M., Chen C., Huang W., Lin J.T., Ke T., Chen L., Tsai M., Wu C. (2008). Benzimidazole/Amine-based compounds capable of ambipolar transport for application in single-layer blue-emitting oleds and as hosts for phosphorescent emitters. Angew. Chem. Int. Ed..

[8] Sim B.R., Kim B.-G., Lee J.K., Do J.Y. (2011). Photovoltaic properties of benzimidazole-derived perylene imides as an n-type material. Thin Solid Films.

[9] Liu Y., Gao Z., Wang Z., Feng C., Shen F., Lu P., Ma Y. (2013). Synthesis and characterization of an imidazole-containing pyrene π-system. Eur. J. Org. Chem..

[10] Chen L., Yin S.-Y., Pan M., Wu K., Wang H.-P., Fan Y.-N., Su C.-Y. (2016). A naked eye colorimetric sensor for alcohol vapor discrimination and amplified spontaneous emission (ASE) from a highly fluorescent excited-state intramolecular proton transfer (ESIPT) molecule. J. Mater. Chem. C.

[11] Nieto C.I., Cabildo P., García M.Á., Claramunt R.M., Alkorta I., Elguero J. (2014). An experimental and theoretical NMR study of NH-benzimidazoles in solution and in the solid state: Proton transfer and tautomerism. Beilstein J. Org. Chem..

[12] Chipem F.A.S., Behera S.K., Krishnamoorthy G. (2014). Excited state proton transfer of 2-(2′-hydroxyphenyl)benzimidazole and its nitrogen substituted analogues in bovine serum albumin. Photochem. Photobiol. Sci..

[13] Forés M., Duran M., Solà M., Orozco M., Luque F.J. (1999). Theoretical evaluation of solvent effects on the conformational and tautomeric equilibriaof 2-(2′-hydroxyphenyl)benzimidazole and on its absorption and fluorescence spectra. J. Phys. Chem. A.

[14] Qin M., Jin D., Che W., Jiang Y., Zhang L., Zhu D., Su Z. (2017). Fluorescence response and detection of Cu^2+^ with 2-(2-hydroxyphenyl)benzimidazole in aqueous medium. Inorg. Chem. Commun..

[15] Vaz P.A.A.M., Rocha J., Silva A.M.S., Guieu S. (2021). Difluoroborate complexes based on 2′-hydroxyphenones as solid-state fluorophores. Dyes Pigment..

[16] Vaz P.A.A.M., Rocha J., Silva A.M.S., Guieu S. (2018). Aggregation-induced emission enhancement of chiral boranils. New J. Chem..

[17] Guieu S., Pinto J., Silva V.L.M., Rocha J., Silva A.M.S. (2015). Synthesis, post-modification and fluorescence properties of boron diketonate complexes. Eur. J. Org. Chem..

[18] Cardona F., Rocha J., Silva A.M.S., Guieu S. (2014). Δ^1^-pyrroline based boranyls: Synthesis, crystal structures and luminescent properties. Dyes Pigment..

[19] Costa L.D., Guieu S., Rocha J., Silva A.M.S., Tomé A.C. (2017). Porphyrin–boron diketonate dyads. New J. Chem..

[20] Cui Y., Wang S. (2006). Diboron and triboron compounds based on linear and star-shaped conjugated ligands with 8-hydroxyquinolate functionality:  impact of intermolecular interaction and boron coordination on luminescence. J. Org. Chem..

[21] Dhanunjayarao K., Mukundam V., Ramesh M., Venkatasubbaiah K. (2014). Synthesis and optical properties of salicylaldimine-based diboron complexes. Eur. J. Inorg. Chem..

[22] Guieu S., Cardona F., Rocha J., Silva A.M.S. (2014). Luminescent bi-metallic fluoroborate derivatives of bulky salen ligands. New J. Chem..

[23] Li G., Lou W., Wang D., Deng C., Zhang Q. (2019). Difluoroboron-enabled thermally activated delayed fluorescence. ACS Appl. Mater. Interfaces.

[24] Azarias C., Budzák Š., Laurent A.D., Ulrich G., Jacquemin D. (2016). Tuning ESIPT fluorophores into dual emitters. Chem. Sci..

[25] Sedgwick A.C., Wu L., Han H.H., Bull S.D., He X.P., James T.D., Sessler J.L., Tang B.Z., Tian H., Yoon J. (2018). Excited-state intramolecular proton-transfer (ESIPT) based fluorescence sensors and imaging agents. Chem. Soc. Rev..

[26] Kim T.-I., Kang H.J., Han G., Chung S.J., Kim Y. (2009). A highly selective fluorescent ESIPT probe for the dual specificity phosphatase MKP-6. Chem. Commun..

[27] Li X., Son Y.-A. (2014). Efficient luminescence from easily prepared fluorine–boron core complexes based on benzothiazole and benzoxazole. Dyes Pigment..

[28] Massue J., Ulrich G., Ziessel R. (2013). Effect of 3,5-disubstitution on the optical properties of luminescent 2-(2′-hydroxyphenyl)benzoxazoles and their borate complexes. Eur. J. Org. Chem..

[29] Massue J., Frath D., Ulrich G., Retailleau P., Ziessel R. (2012). Synthesis of luminescent 2-(2′-hydroxyphenyl)benzoxazole (hbo) borate complexes. Org. Lett..

[30] Frath D., Massue J., Ulrich G., Ziessel R. (2014). Luminescent materials: Locking π-conjugated and heterocyclic ligands with boron(III). Angew. Chem. Int. Ed..

[31] Mukundam V., Dhanunjayarao K., Samal S., Venkatasubbaiah K. (2017). Variation of para substituent on 2-phenol of tetraaryl-substituted imidazole-boron difluoride complexes: Synthesis, characterization, and photophysical properties. Asian J. Org. Chem..

[32] Benelhadj K., Massue J., Retailleau P., Ulrich G., Ziessel R. (2013). 2-(2′-Hydroxyphenyl)benzimidazole and 9,10-phenanthroimidazole chelates and borate complexes: Solution- and solid-state emitters. Org. Lett..

[33] Dhanunjayarao K., Mukundam V., Venkatasubbaiah K. (2016). tetracoordinate imidazole-based boron complexes for the selective detection of picric acid. Inorg. Chem..

[34] Xu H., Xu Z.-F., Yue Z.-Y., Yan P.-F., Wang B., Jia L.-W., Li G.-M., Sun W.-B., Zhang J.-W. (2008). A novel deep blue-emitting znii complex based on carbazole-modified 2-(2-hydroxyphenyl)benzimidazole: Synthesis, bright electroluminescence, and substitution effect on photoluminescent, thermal, and electrochemical properties. J. Phys. Chem. C.

[35] Esparza-Ruiz A., Peña-Hueso A., Nöth H., Flores-Parra A., Contreras R. (2009). Boron coordination compounds derived from 2-phenyl-benzimidazole and 2-phenyl-benzotriazole bidentate ligands. J. Organomet. Chem..

[36] Bolton O., Lee K., Kim H.-J., Lin K.Y., Kim J. (2011). Activating efficient phosphorescence from purely organic materials by crystal design. Nat. Chem..

[37] Ouyang J., Ouyang C., Fujii Y., Nakano Y., Shoda T., Nagano T. (2004). Synthesis and fluorescent properties of 2-(1H-benzimidazol-2-yl)-phenol derivatives. J. Heterocycl. Chem..

[38] Alaqeel S.I. (2017). Synthetic approaches to benzimidazoles from o-phenylenediamine: A literature review. J. Saudi Chem. Soc..

[39] Resch-Genger U., Rurack K. (2013). Determination of the photoluminescence quantum yield of dilute dye solutions (IUPAC Technical Report). Pure Appl. Chem..

[40] Manojai N., Daengngern R., Kerdpol K., Ngaojampa C., Kungwan N. (2017). Heteroatom effect on photophysical properties of 2-(2′-hydroxyphenyl)benzimidazole and its derivatives as fluorescent dyes: A TD-DFT study. J. Lumin..

[41] Akutsu K., Mori S., Shinmei K., Iwase H., Nakano Y., Fujii Y. (2016). Investigation of substitution effect on fluorescence properties of Zn^2+^-selective ratiometric fluorescent compounds: 2-(2-Hydroxyphenyl)benzimidazole derivatives. Talanta.

[42] Padalkar V.S., Ramasami P., Sekar N. (2014). A comprehensive spectroscopic and computational investigation of intramolecular proton transfer in the excited states of 2-(2-hydroxyphenyl) benzoxazole and its derivatives. J. Lumin..

[43] Chipem F.A.S., Dash N., Krishnamoorthy G. (2011). Role of nitrogen substitution in phenyl ring on excited state intramolecular proton transfer and rotamerism of 2-(2-hydroxyphenyl)benzimidazole: A theoretical study. J. Chem. Phys..

[44] Wang R., Liu D., Xu K., Li J. (2009). Substituent and solvent effects on excited state intramolecular proton transfer in novel 2-(2′-hydroxyphenyl)benzothiazole derivatives. J. Photochem. Photobiol. A.

[45] Fontes L.F.B., Nunes da Silva R., Silva A.M.S., Guieu S. (2020). Unsymmetrical 2,4,6-triarylpyridines as versatile scaffolds for deep-blue and dual emission fluorophores. ChemPhotoChem.

[46] Chen K.-Y., Hsieh C.-C., Cheng Y.-M., Lai C.-H., Chou P.-T. (2006). Extensive spectral tuning of the proton transfer emission from 550 to 675 nm via a rational derivatization of 10-hydroxybenzo[*h*]quinoline. Chem. Commun..

[47] Foley S., Berberan-Santos M.N., Fedorov A., Bensasson R.V., Leach S., Gigante B. (2001). Effect of halogenated compounds on the photophysics of C70 and a monoadduct of C70: Some implications on optical limiting behaviour. Chem. Phys..

[48] Zhang Z., Zhang H., Jiao C., Ye K., Zhang H., Zhang J., Wang Y. (2015). 2-(2-hydroxyphenyl)benzimidazole-based four-coordinate boron-containing materials with highly efficient deep-blue photoluminescence and electroluminescence. Inorg. Chem..

[49] Yang X., Xu X., Zhou G. (2015). Recent advances of the emitters for high performance deep-blue organic light-emitting diodes. J. Mater. Chem. C.

[50] (2004). ITU-R Recommendation BT.1700, Characteristics of Composite Video Signals for Conventional Analogue Television Systems. https://www.itu.int/rec/R-REC-BT.1700-0-200502-I/en.

