# Supplementary material for: Benzimidazole-Based N,O Boron Complexes as Deep Blue Solid-State Fluorophores
Source: Materials (Basel). 2021 Jul 31;14(15):4298. doi: 10.3390/ma14154298 (PMC8348718; doi:10.3390/ma14154298)
Supplement: Supplementary file 1 [file materials-14-04298-s001.zip › materials-1293823-supplementary.pdf]

Supporting Information for

Benzimidazole-based N,O boron complexes as deep blue solid-state fluorophores

Patricia A. A. M. Vaz,[a,b] João Rocha,[b] Artur M. S. Silva[a] and Samuel Guieu,[a,b,*]

[a] LAQV-REQUIMTE, Department of Chemistry, University of Aveiro, 3810-193 Aveiro, Portugal.

[b] CICECO, Department of Chemistry, University of Aveiro, 3810-193 Aveiro, Portugal.

*To whom correspondence should be addressed. E-mail: sguieu@ua.pt

NMR spectra..2
Absorption, emission and excitation spectra in solution..18
Excitation and emission spectra in solid state..22

NMR spectra

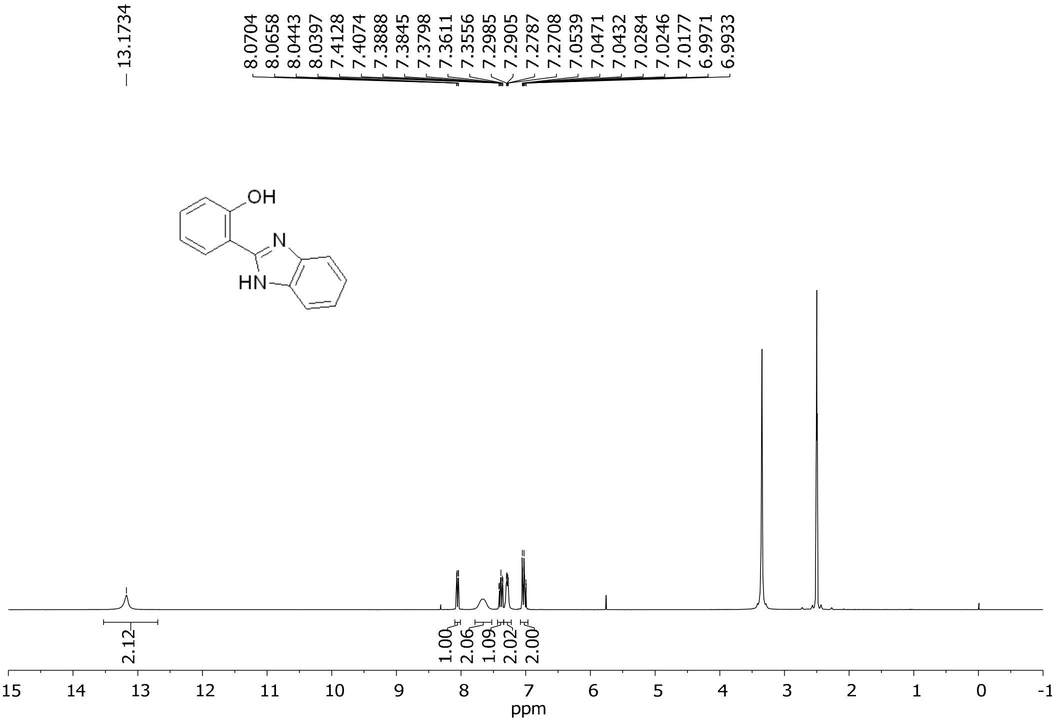

Figure S1 - ^{1}H NMR spectrum of 2-(1*H*-benzo[*d*]imidazol-2-yl)phenol **7a** in DMSO-*d*$_6$.

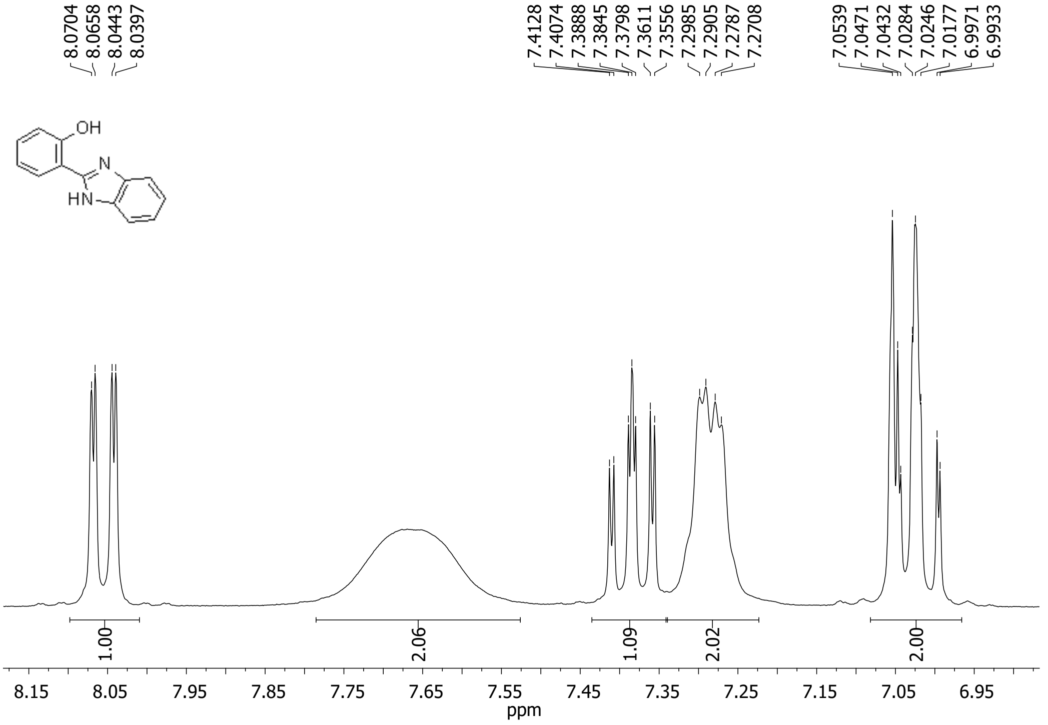

Figure S2– ^{1}H NMR spectrum expansion of 2-(1*H*-benzo[*d*]imidazol-2-yl)phenol **7a** in DMSO-*d*$_6$.

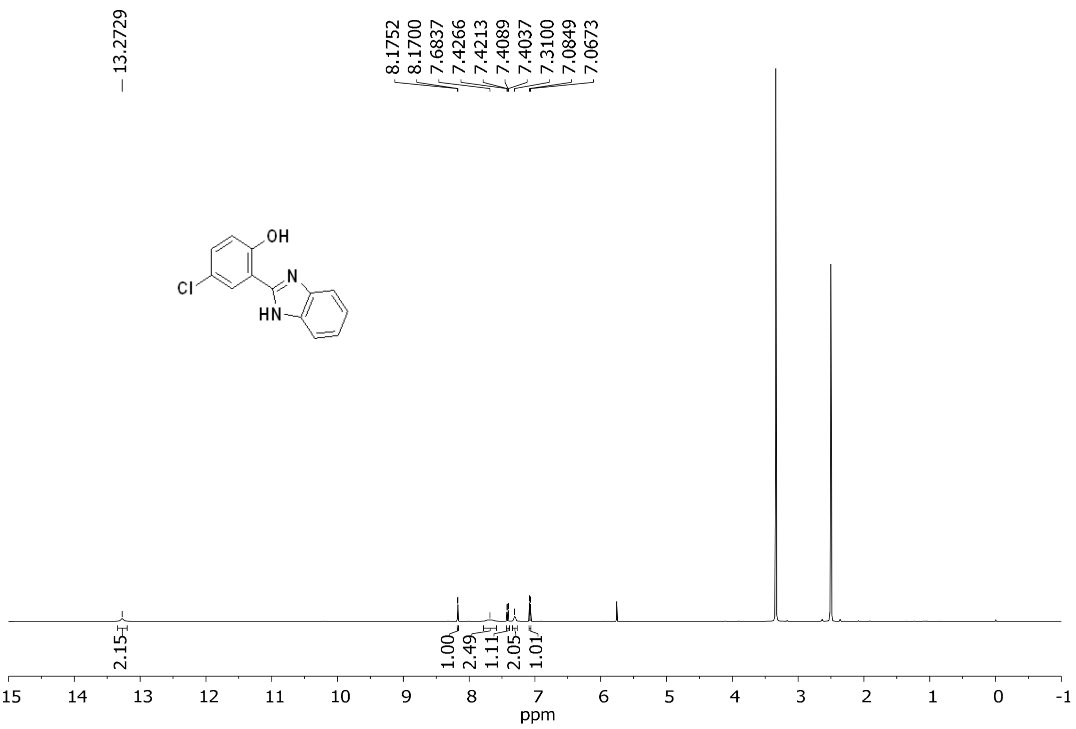

Figure S3- ^{1}H NMR spectrum of 2-(1*H*-benzo[*d*]imidazol-2-yl)-4-chlorophenol **7b** in DMSO-d_6.

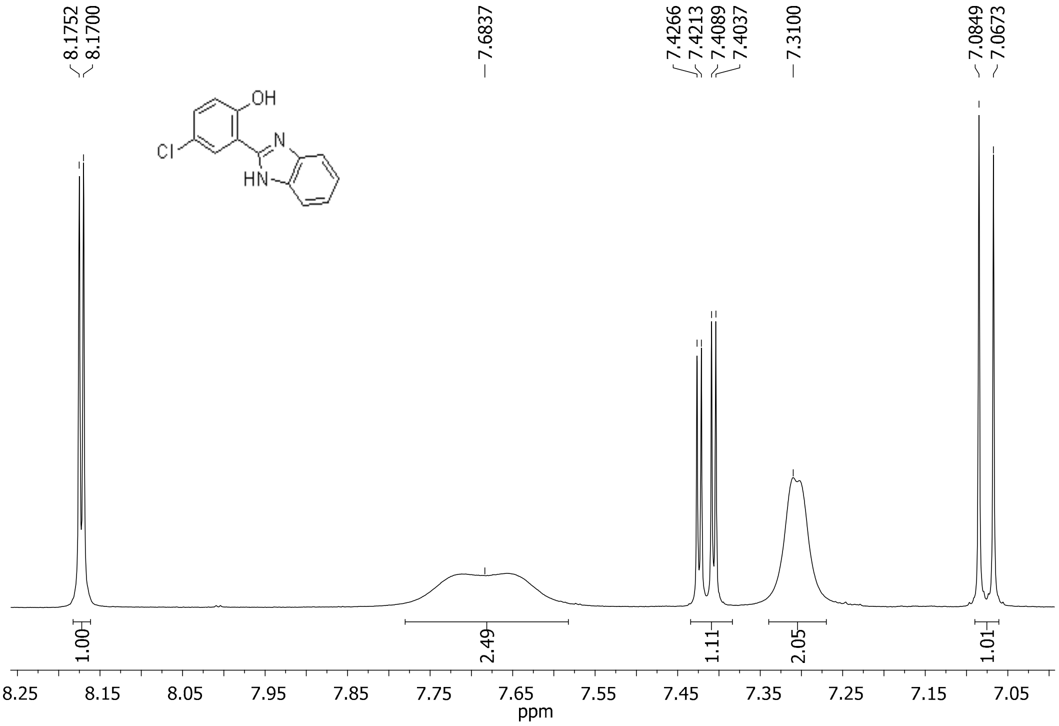

Figure S4- ^{1}H NMR spectrum expansion of 2-(1*H*-benzo[*d*]imidazol-2-yl)-4-chlorophenol **7b** in DMSO-d_6.

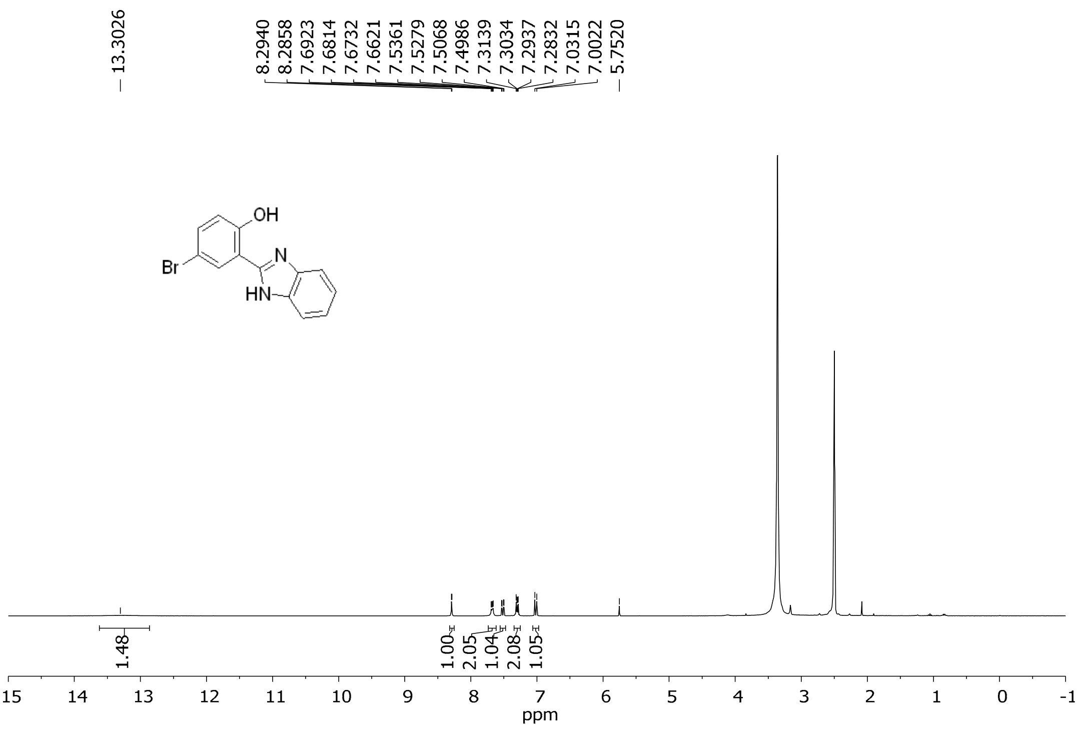

Figure S5- ^{1}H NMR spectrum of 2-(1*H*-benzo[*d*]imidazol-2-yl)-4-bromophenol **7c** in DMSO-d_6.

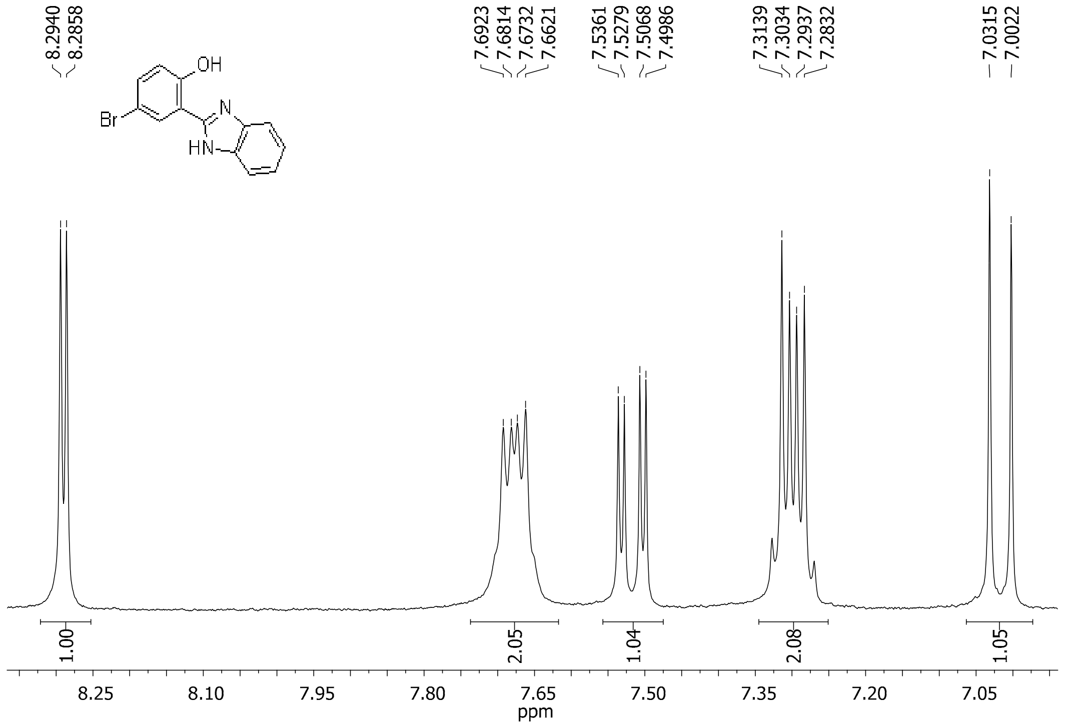

Figure S6-. ^{1}H NMR spectrum expansion of 2-(1*H*-benzo[*d*]imidazol-2-yl)-4-bromophenol **7c** in DMSO-d_6.

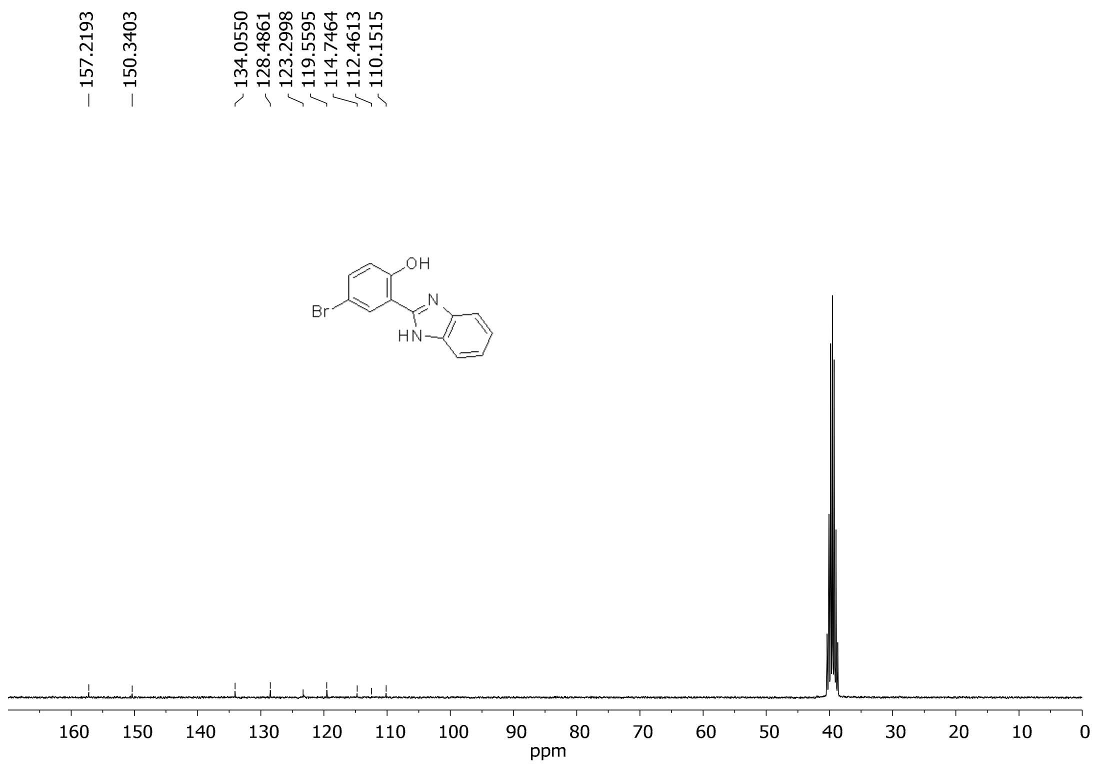

Figure S7– ^{13}C NMR spectrum of 2-(1*H*-benzo[*d*]imidazol-2-yl)-4-bromophenol **7c** in DMSO-d_6.

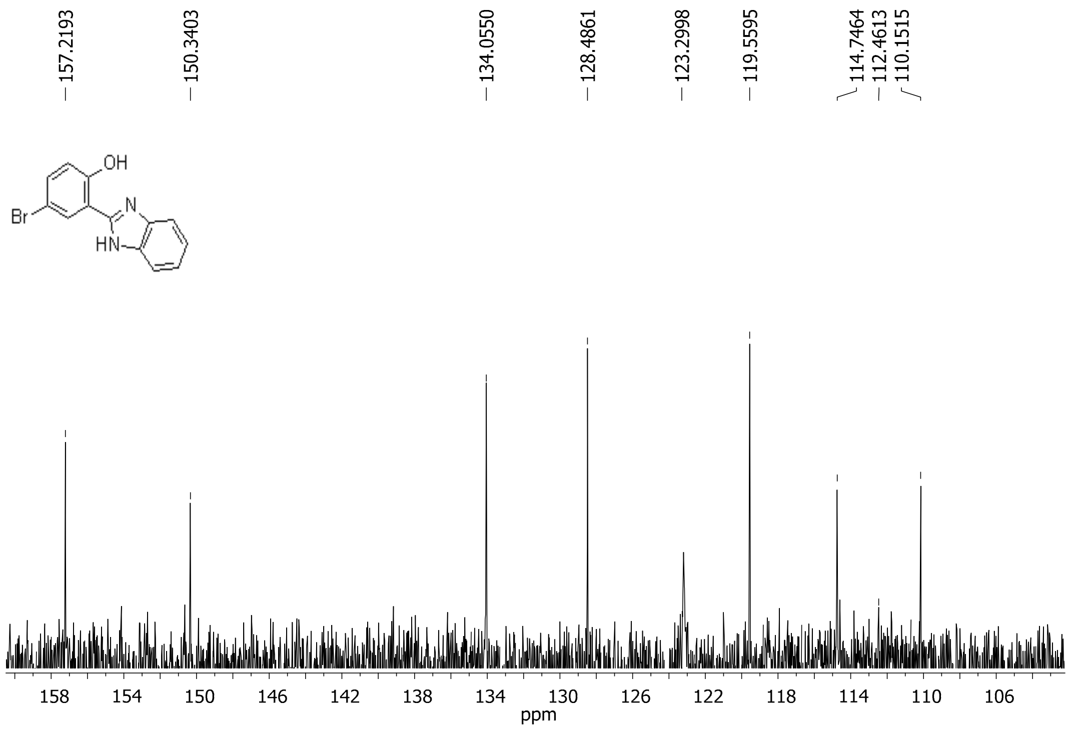

Figure S8- ^{13}C NMR spectrum expansion of 2-(1*H*-benzo[*d*]imidazol-2-yl)-4-bromophenol **7c** in DMSO-d_6.

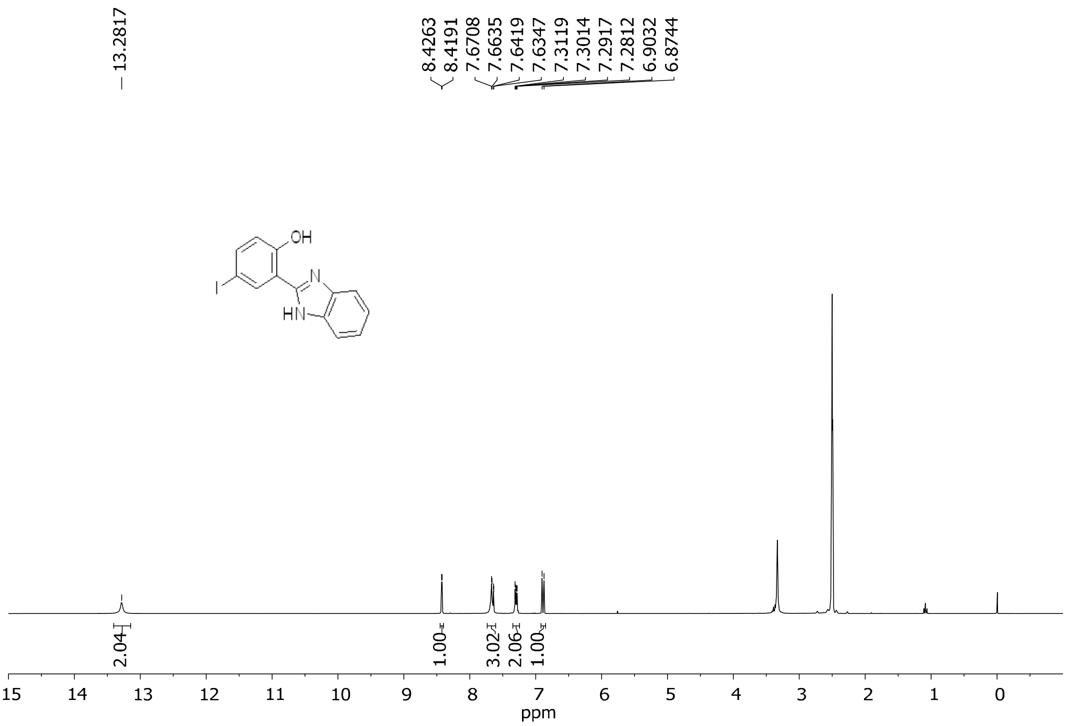

Figure S9- ^{1}H NMR spectrum of 2-(1*H*-benzo[*d*]imidazol-2-yl)-4-iodophenol **7d** in DMSO-*d*$_6$.

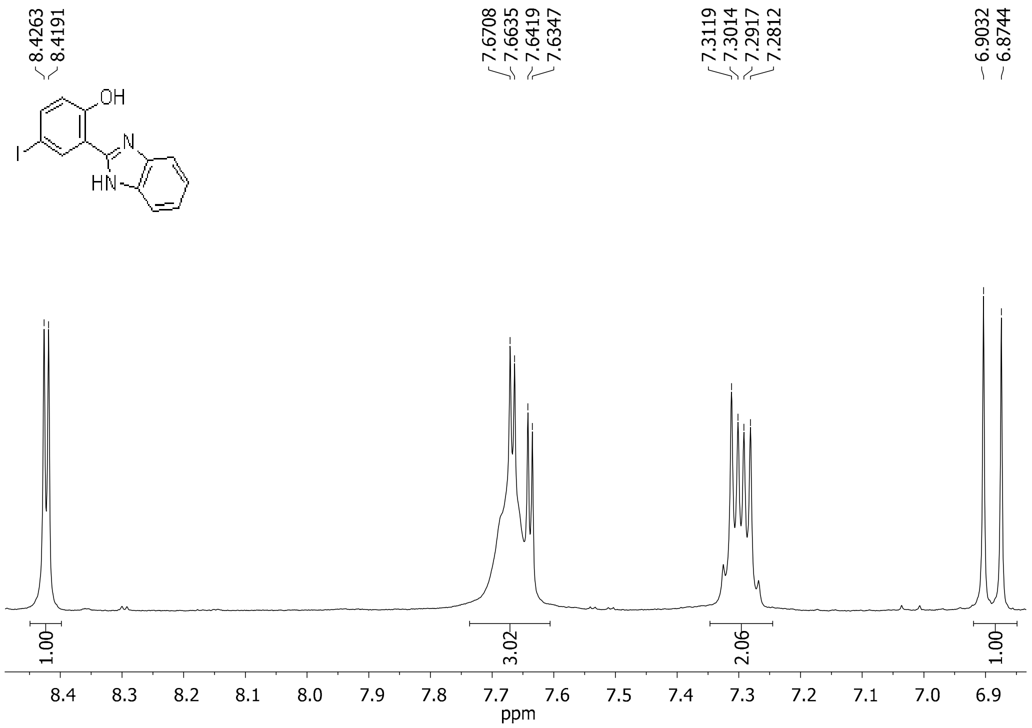

Figure S10- ^{1}H NMR spectrum expansion of 2-(1*H*-benzo[*d*]imidazol-2-yl)-4-iodophenol **7d** in DMSO-*d*$_6$.

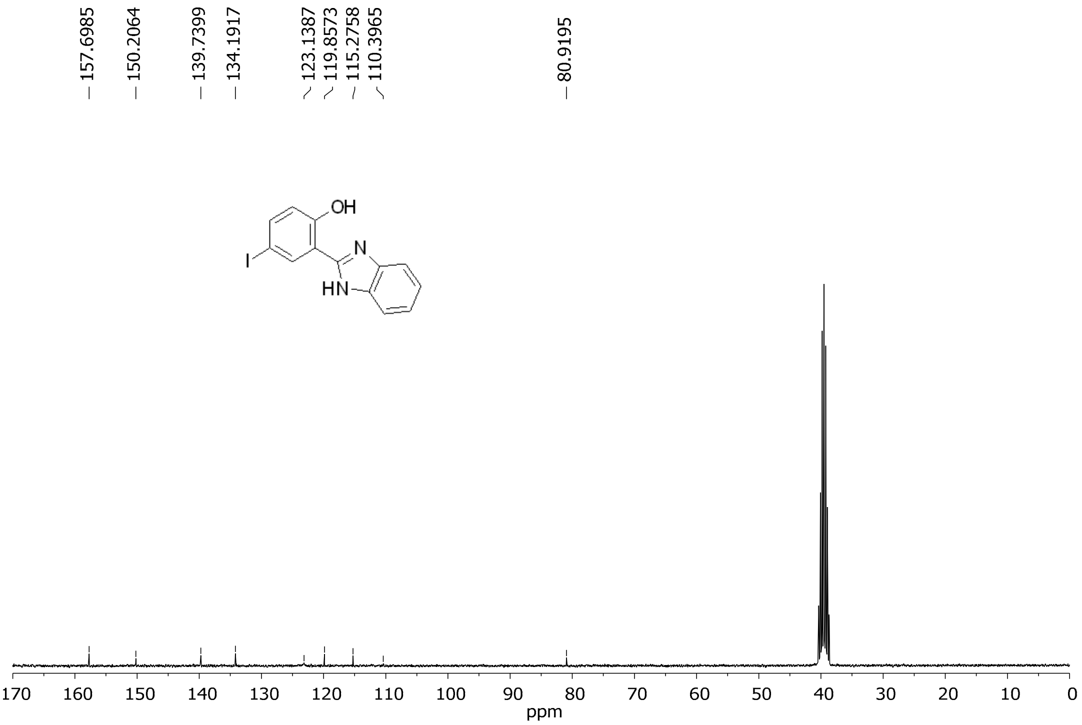

Figure S11– ^{13}C NMR spectrum of 2-(1*H*-benzo[*d*]imidazol-2-yl)-4-iodophenol **7d** in DMSO-d_6.

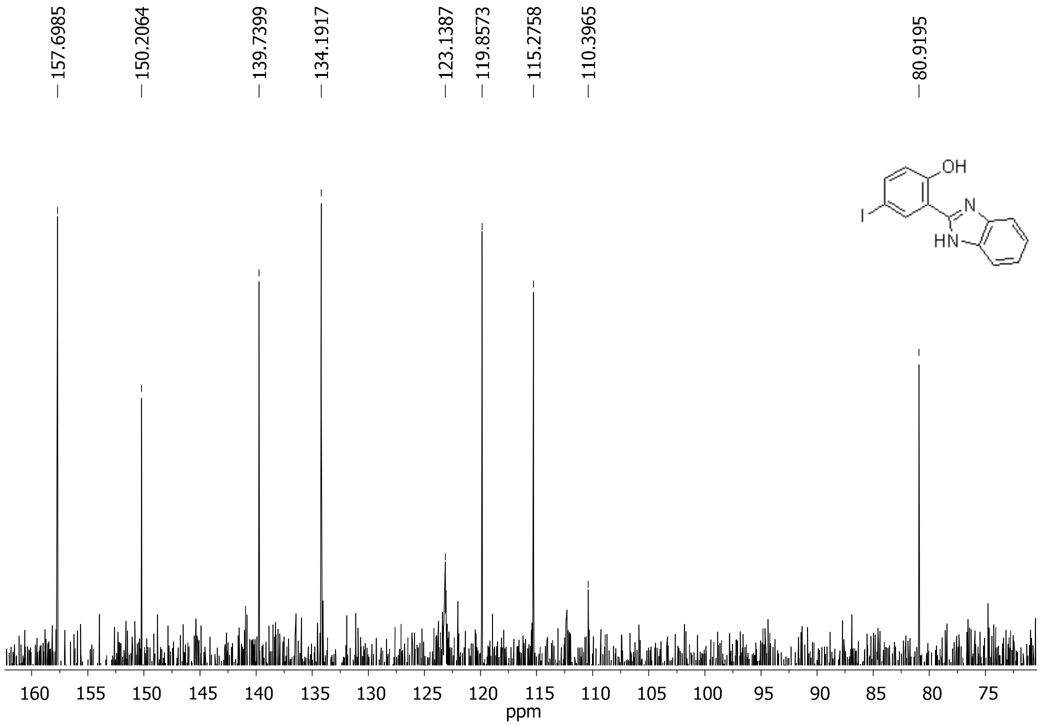

Figure S12- ^{13}C NMR spectrum expansion of 2-(1*H*-benzo[*d*]imidazol-2-yl)-4-iodophenol **7d** in DMSO-d_6.

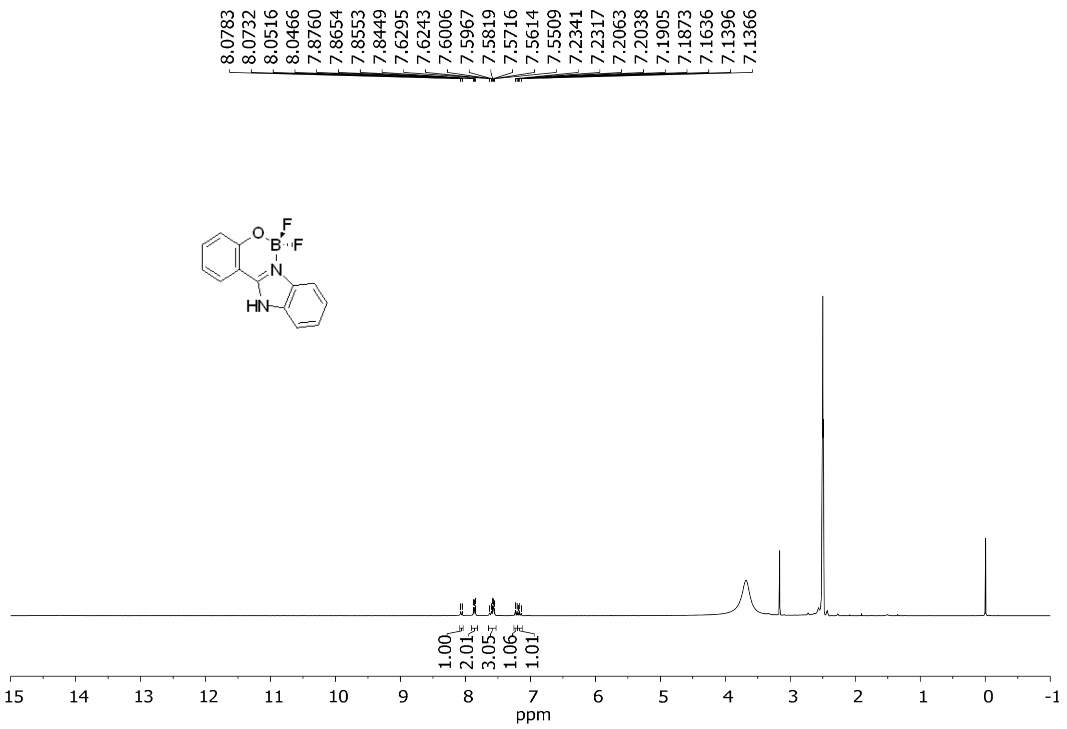

Figure S13 – ^{1}H NMR spectrum of 6,6-difluoro-6,12-dihydrobenzo[*e*]benzo[4,5]imidazo[1,2-*c*][1,3,2]oxazaborinin-7-ium-6-uide **8a** in DMSO-d_6.

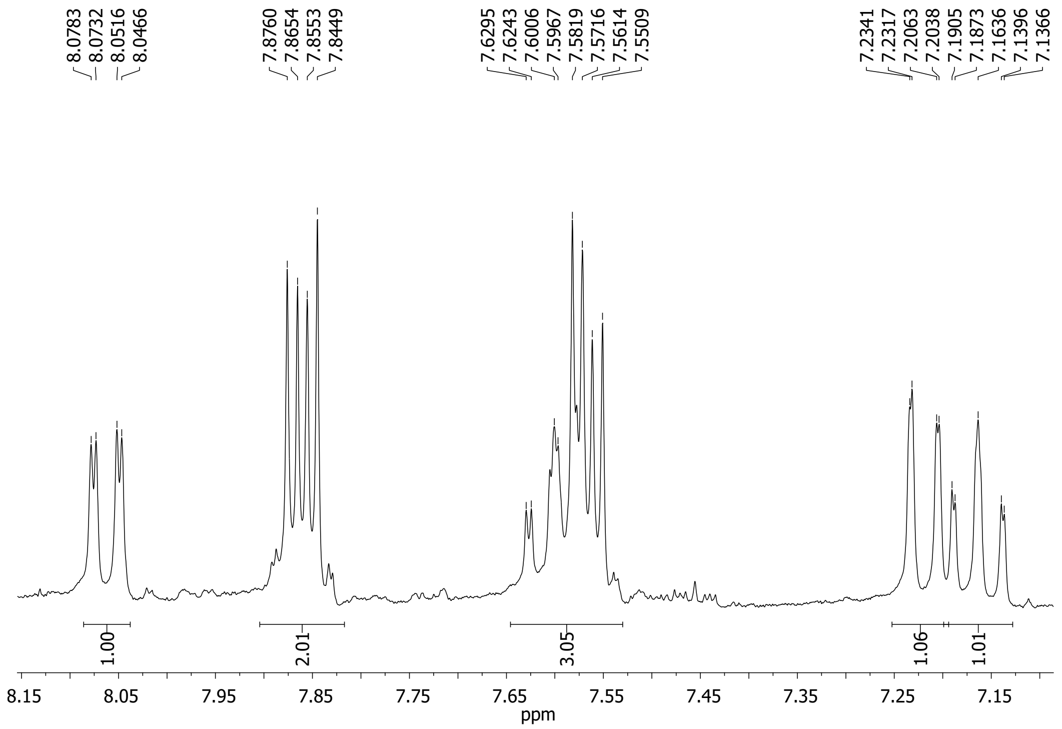

Figure S14 - ^{1}H NMR spectrum expansion of 6,6-difluoro-6,12-dihydrobenzo[*e*]benzo[4,5]imidazo[1,2-*c*][1,3,2]oxazaborinin-7-ium-6-uide **8a** in DMSO-d_6.

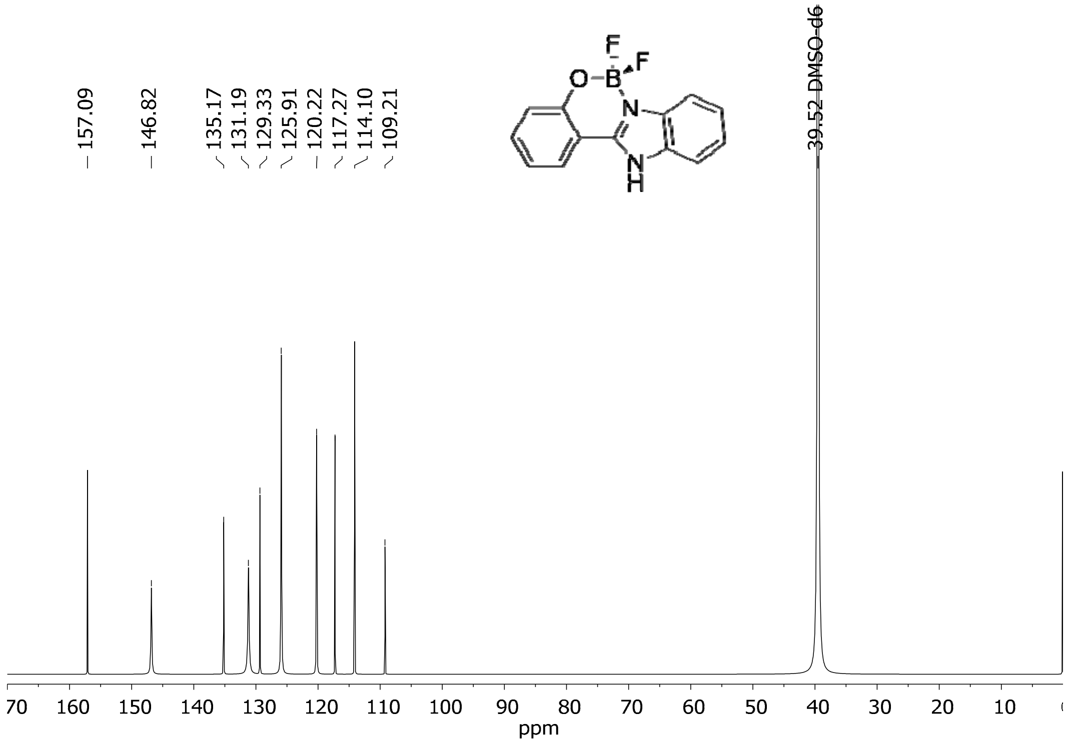

Figure S15 – ^{13}C NMR spectrum expansion of 6,6-difluoro-6,12-dihydrobenzo[*e*]benzo[4,5]imidazo[1,2-*c*][1,3,2]oxazaborinin-7-ium-6-uide **8a** in DMSO-d_6.

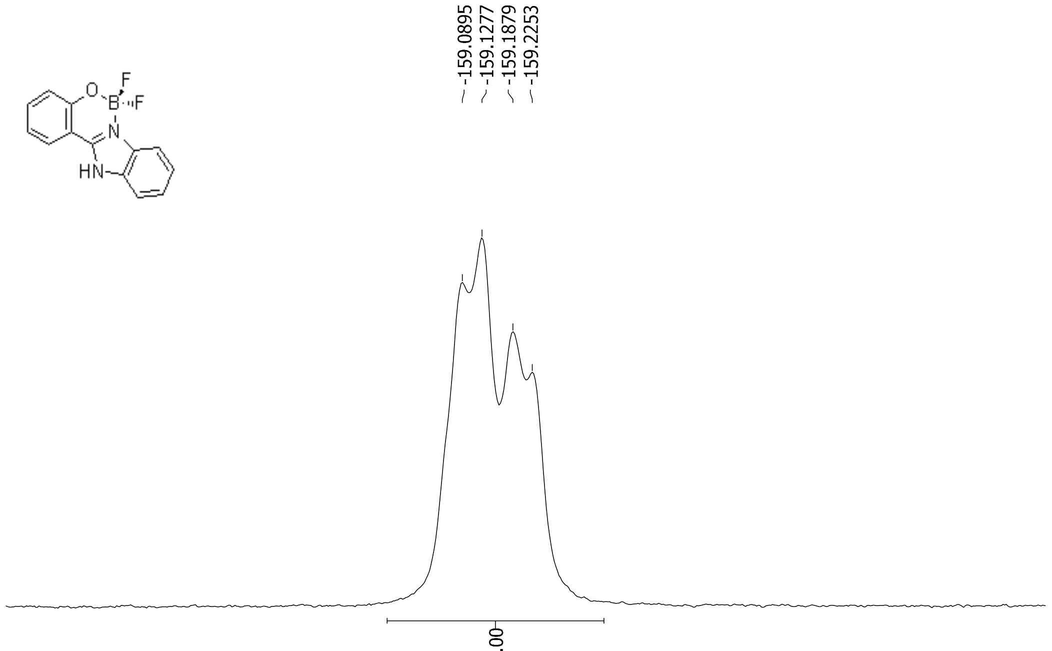

Figure S16 -^{19}F NMR spectrum of 6,6-difluoro-6,12-dihydrobenzo[*e*]benzo[4,5]imidazo[1,2-*c*][1,3,2]oxazaborinin-7-ium-6-uide **8a** in DMSO-d_6.

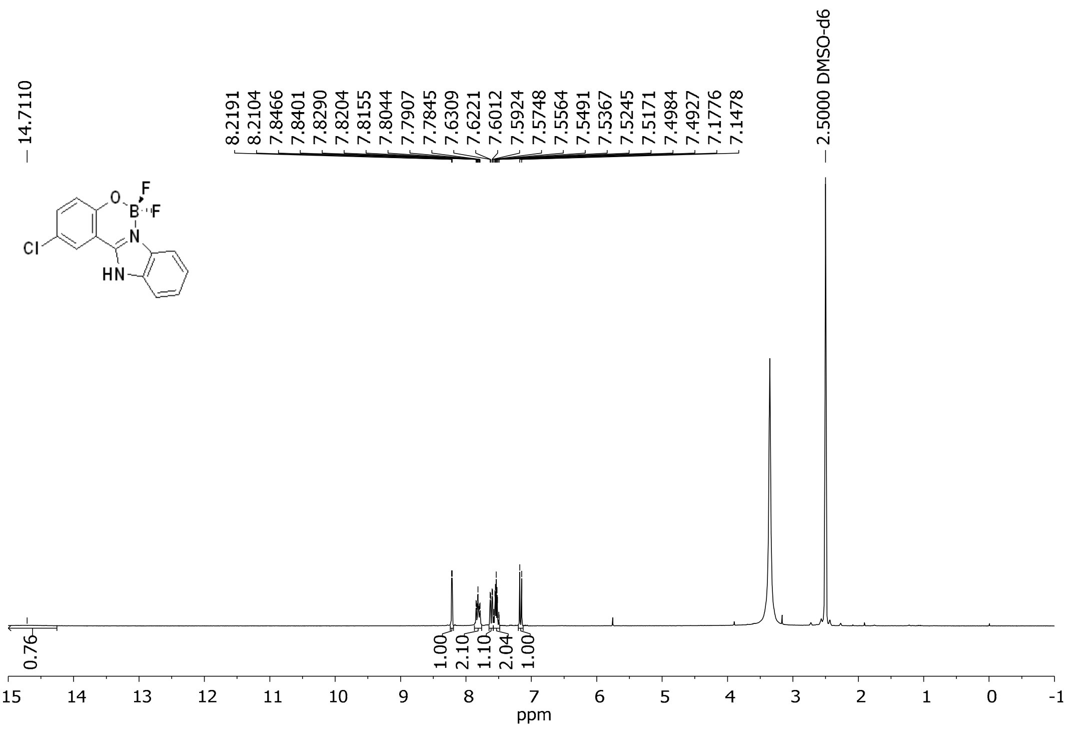

Figure S17-¹H NMR spectrum of 2-chloro-6,6-difluoro-6,12-dihydrobenzo[*e*]benzo[4,5]imidazo[1,2-*c*][1,3,2]oxazaborinin-7-ium-6-uide **8b** in DMSO-d_6.

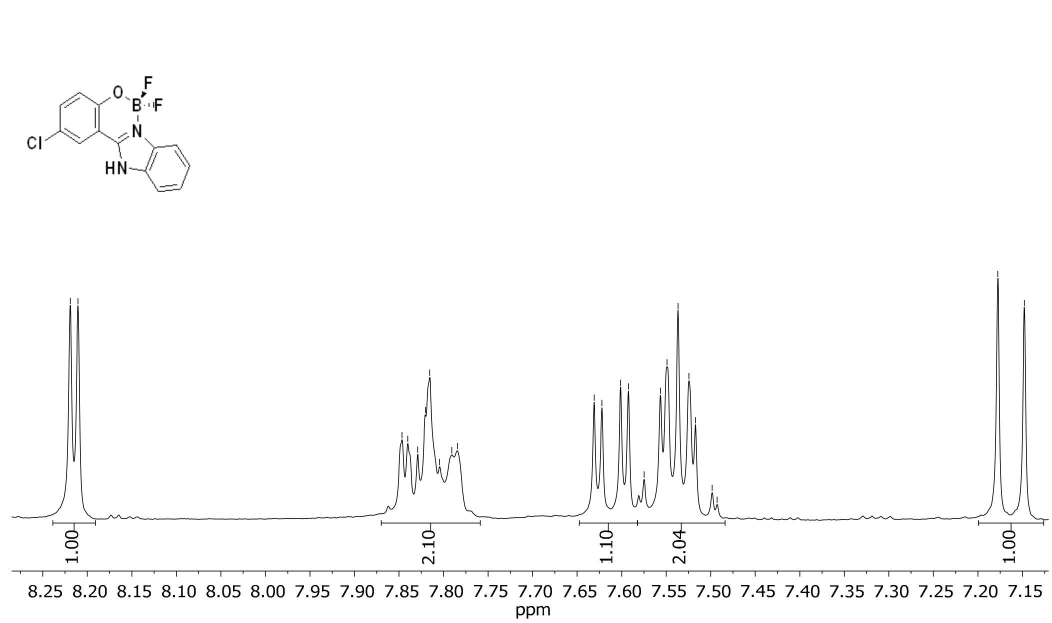

Figure S18-¹H NMR spectrum expansion of 2-chloro-6,6-difluoro-6,12-dihydrobenzo[*e*]benzo[4,5]imidazo[1,2-*c*][1,3,2]oxazaborinin-7-ium-6-uide **8b** in DMSO-d_6.

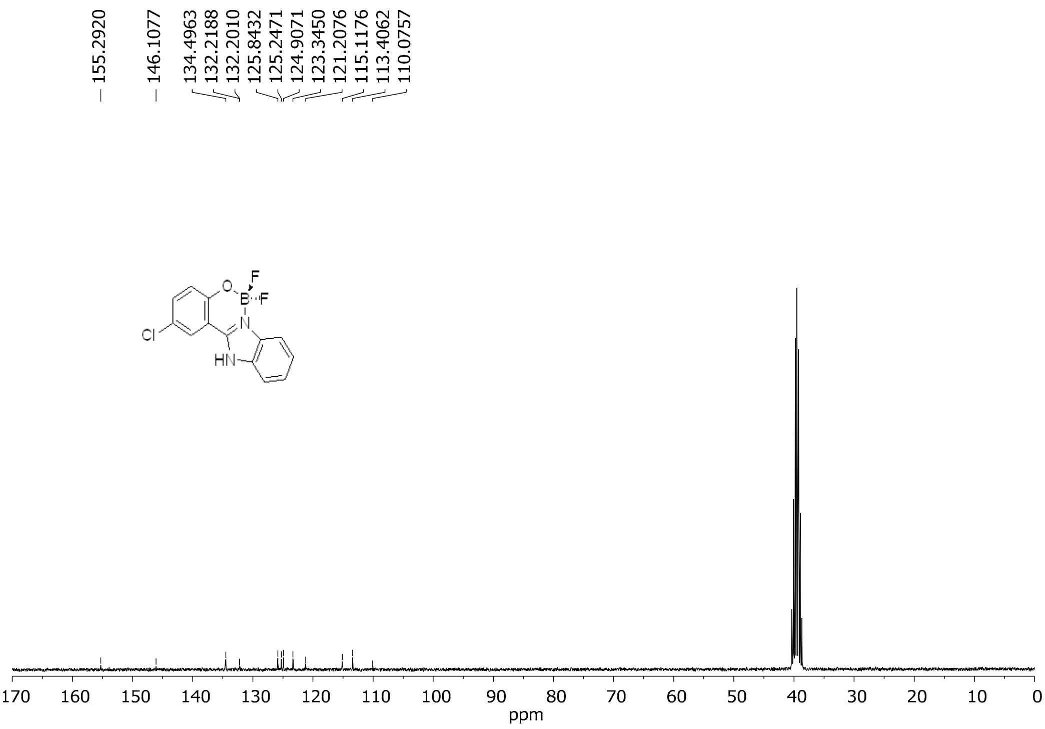

Figure S19– ${}^{13}C$ NMR spectrum of 2-chloro-6,6-difluoro-6,12-dihydrobenzo[*e*]benzo[4,5]imidazo[1,2-*c*][1,3,2]oxazaborinin-7-ium-6-uide **8b** in DMSO-d_6.

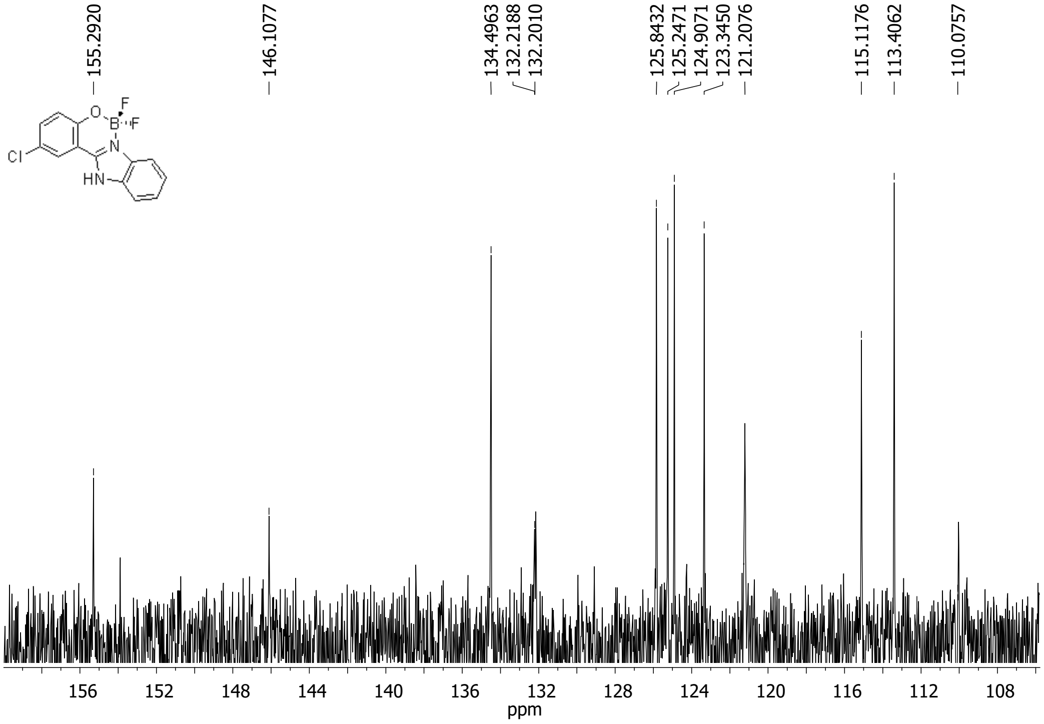

Figure S20 - ${}^{13}C$ NMR spectrum expansion of 2-chloro-6,6-difluoro-6,12-dihydrobenzo[*e*]benzo[4,5]imidazo[1,2-*c*][1,3,2]oxazaborinin-7-ium-6-uide **8b** in DMSO-d_6.

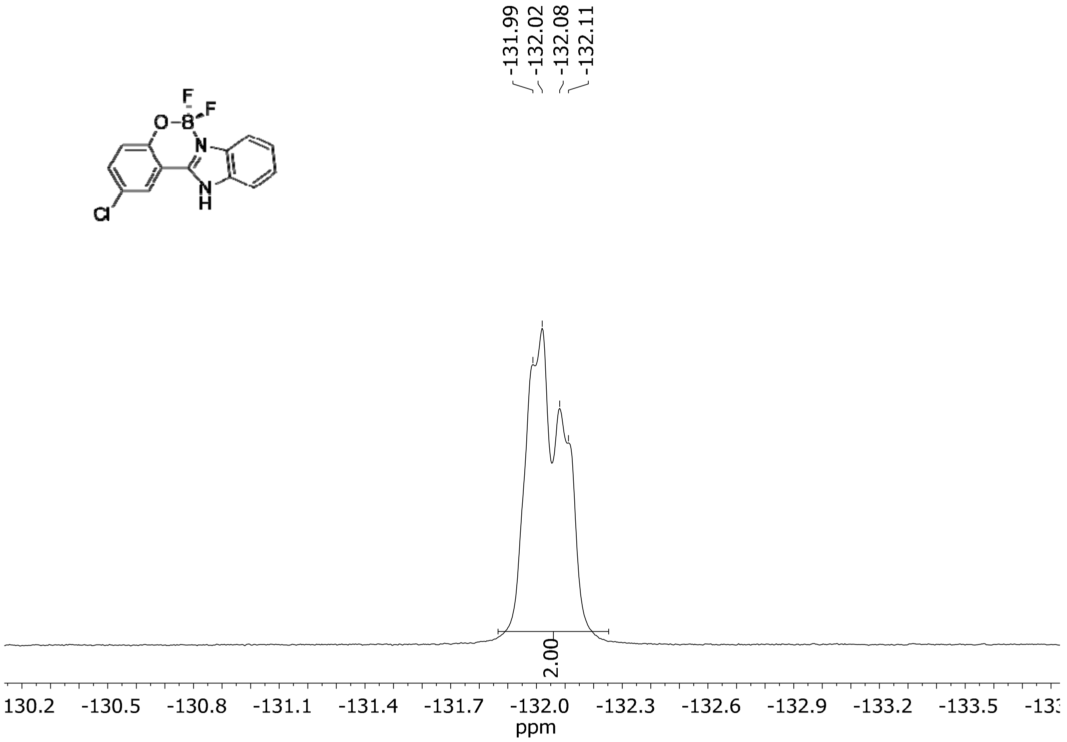

Figure S21 - ^{19}F NMR spectrum of 2-chloro-6,6-difluoro-6,12-dihydrobenzo[*e*]benzo[4,5]imidazo[1,2-*c*][1,3,2]oxazaborinin-7-ium-6-uide **8b** in DMSO-d_6.

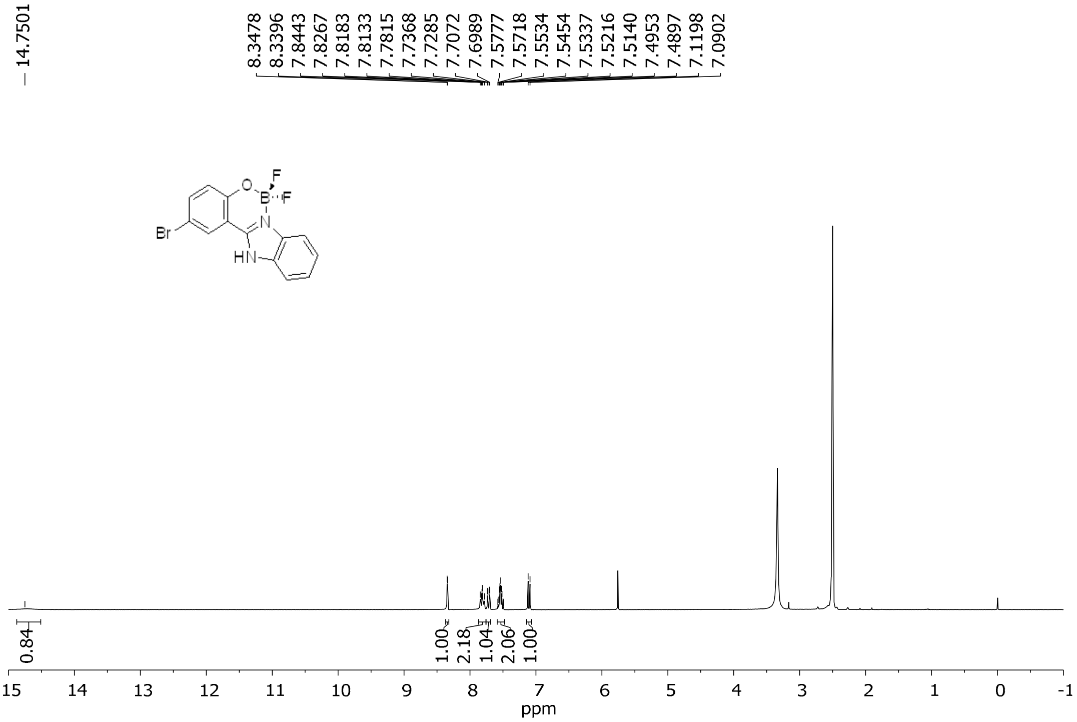

Figure S22- ^{1}H NMR spectrum of 2-bromo-6,6-difluoro-6,12-dihydrobenzo[*e*]benzo[4,5]imidazo[1,2-*c*][1,3,2]oxazaborinin-7-ium-6-uide **8c** in DMSO-d_6.

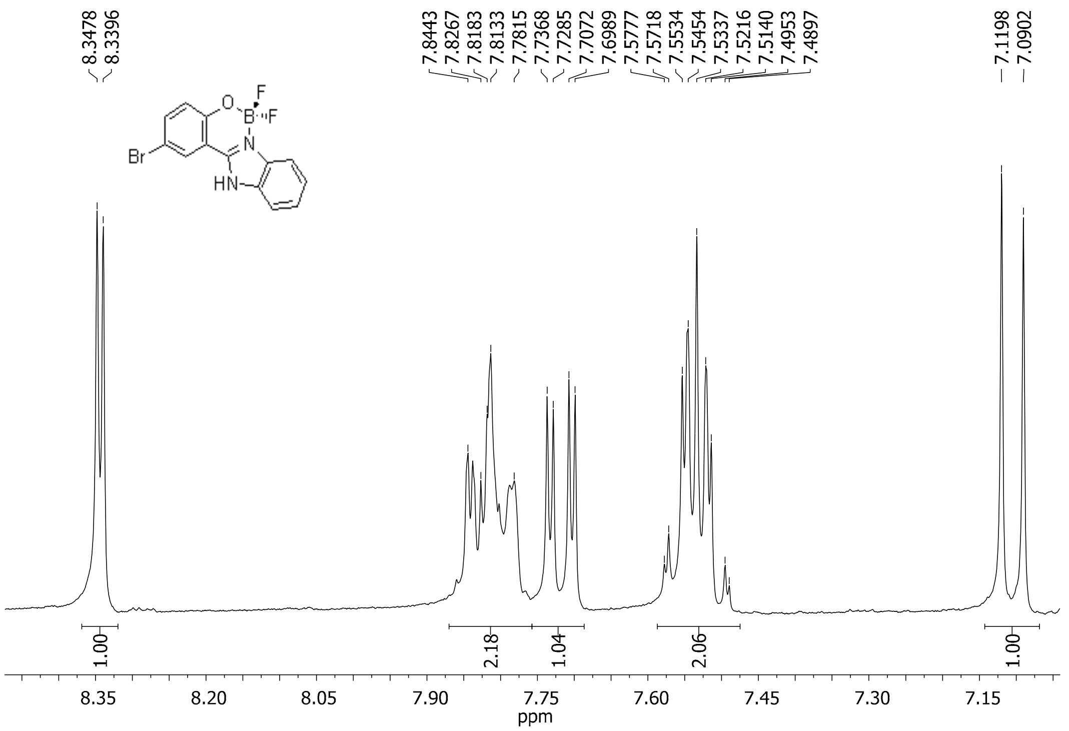

Figure S23 - ^{1}H NMR spectrum expansion of 2-bromo-6,6-difluoro-6,12-dihydrobenzo[*e*]benzo[4,5]imidazo[1,2-*c*][1,3,2]oxazaborinin-7-ium-6-uide **8c** in DMSO-d_6.

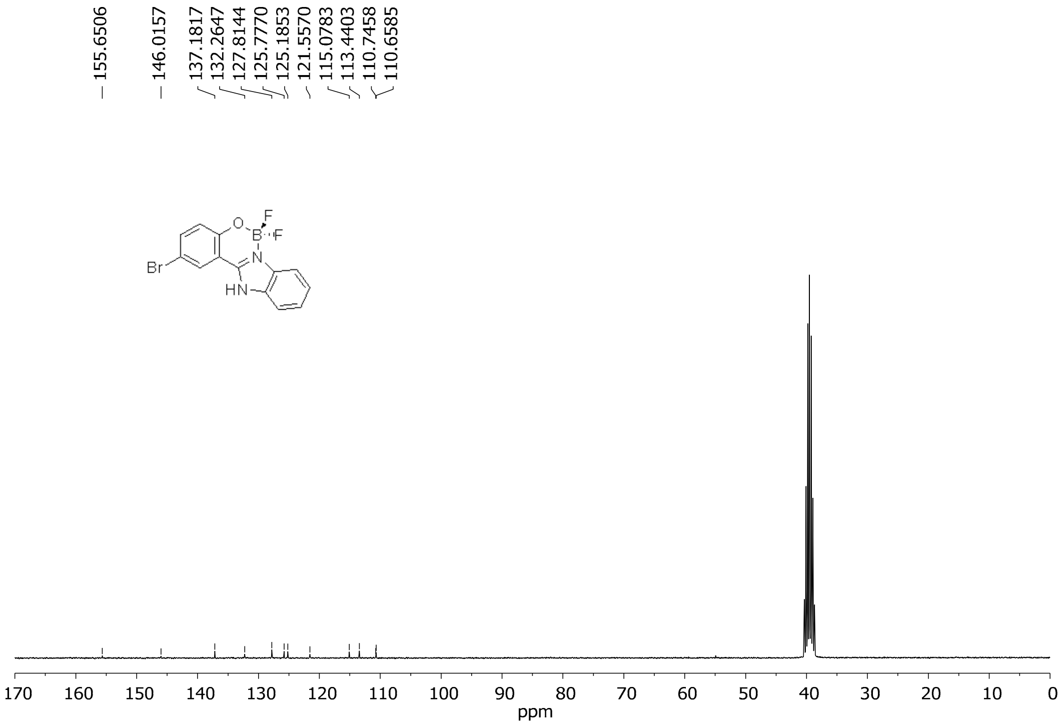

Figure S24 - ^{13}C NMR spectrum expansion of 2-bromo-6,6-difluoro-6,12-dihydrobenzo[*e*]benzo[4,5]imidazo[1,2-*c*][1,3,2]oxazaborinin-7-ium-6-uide **8c** in DMSO-d_6.

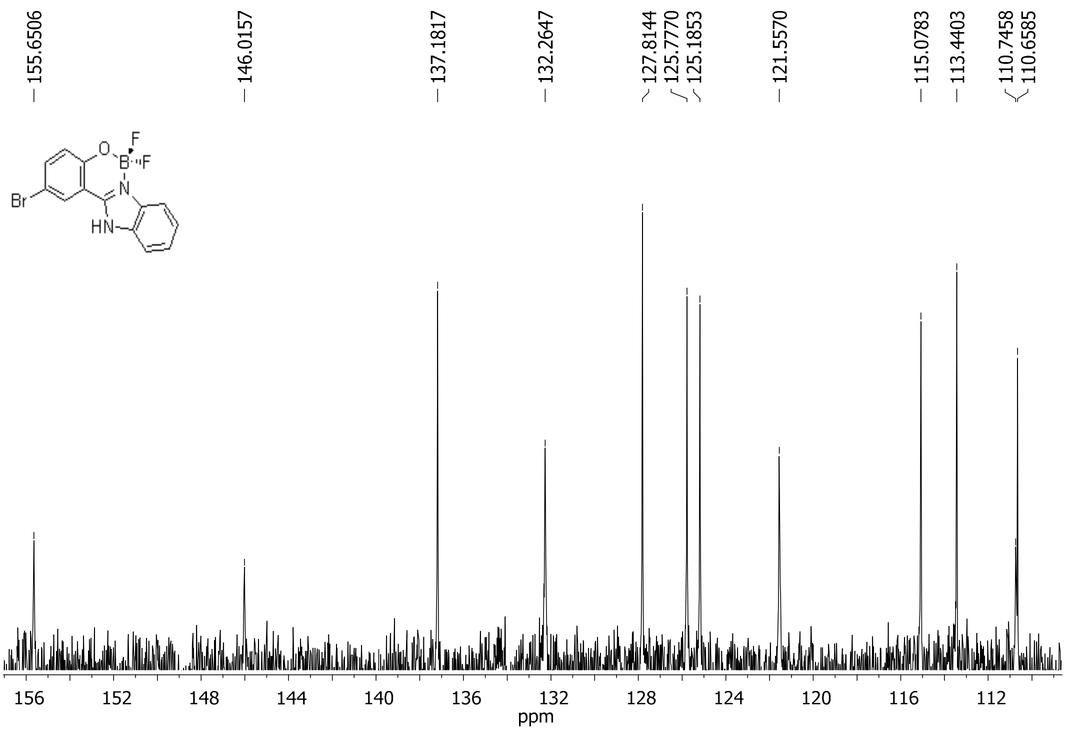

Figure S25– ^{13}C NMR spectrum expansion of 2-bromo-6,6-difluoro-6,12-dihydrobenzo[*e*]benzo[4,5]imidazo[1,2-*c*][1,3,2]oxazaborinin-7-ium-6-uide **8c** in DMSO-d_6.

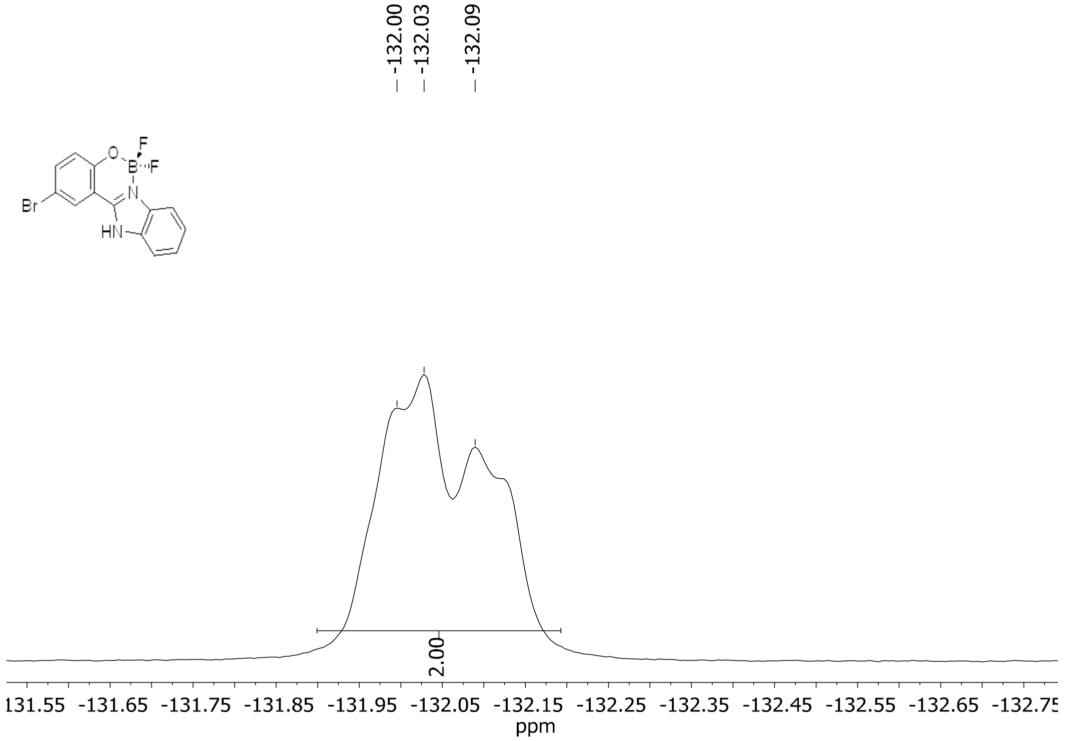

Figure S26– ^{19}F NMR spectrum expansion of 2-bromo-6,6-difluoro-6,12-dihydrobenzo/*e*]benzo[4,5]imidazo[1,2-*c*][1,3,2]oxazaborinin-7-ium-6-uide **8c** in DMSO-d_6.

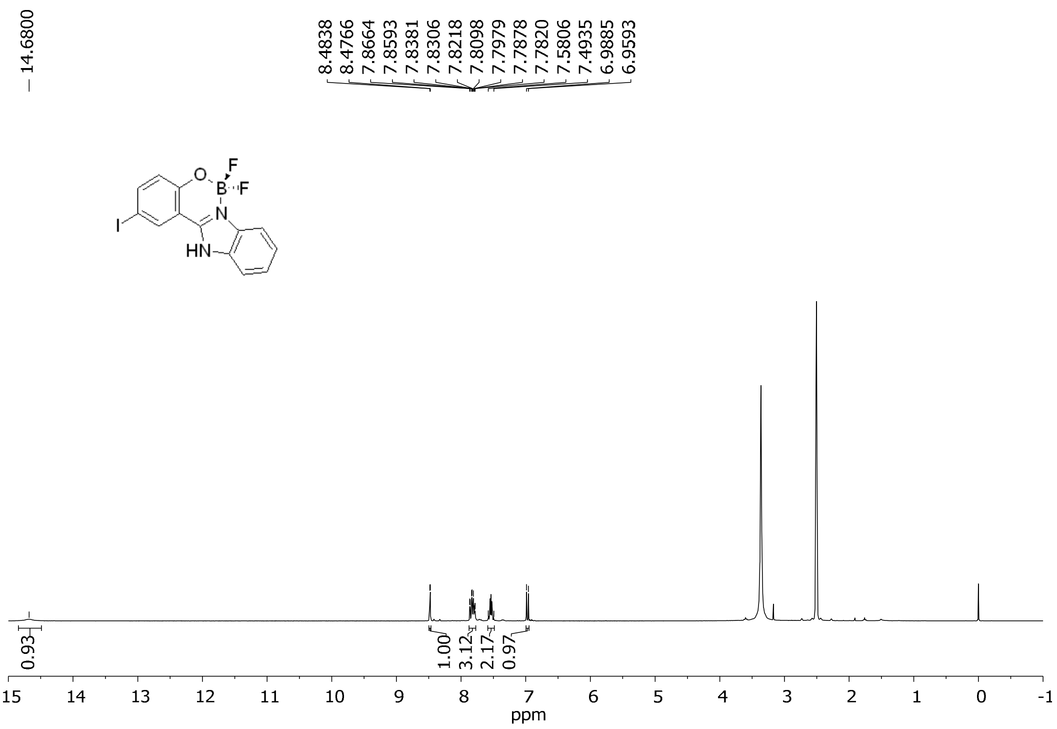

Figure S27 - ^{1}H NMR spectrum of 2-iodo-6,6-difluoro-6,12-dihydrobenzo[*e*]benzo[4,5]imidazo[1,2-*c*][1,3,2]oxazaborinin-7-ium-6-uide **8d** in DMSO-d_6.

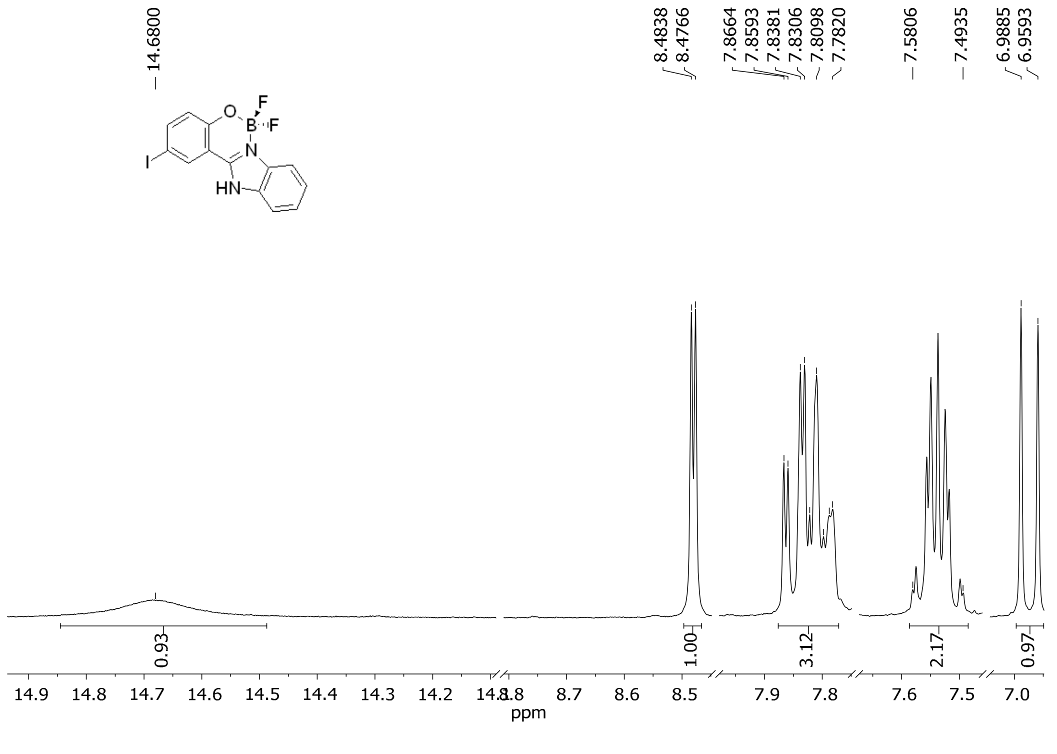

Figure S28 - ^{1}H NMR spectrum expansion of 2-iodo-6,6-difluoro-6,12-dihydrobenzo[*e*]benzo[4,5]imidazo[1,2-*c*][1,3,2]oxazaborinin-7-ium-6-uide **8d** in DMSO-d_6.

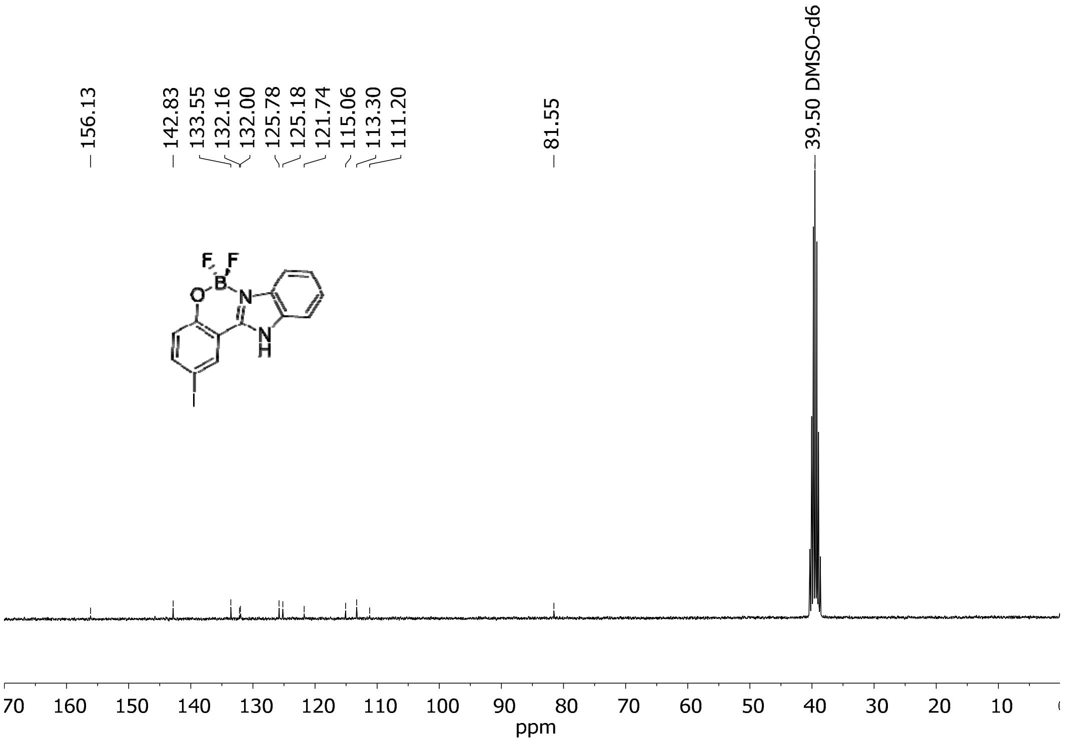

Figure S29 - ^{13}C NMR spectrum of 2-iodo-6,6-difluoro-6,12-dihydrobenzo[*e*]benzo[4,5]imidazo[1,2-*c*][1,3,2]oxazaborinin-7-ium-6-uide **8d** in DMSO-d_6.

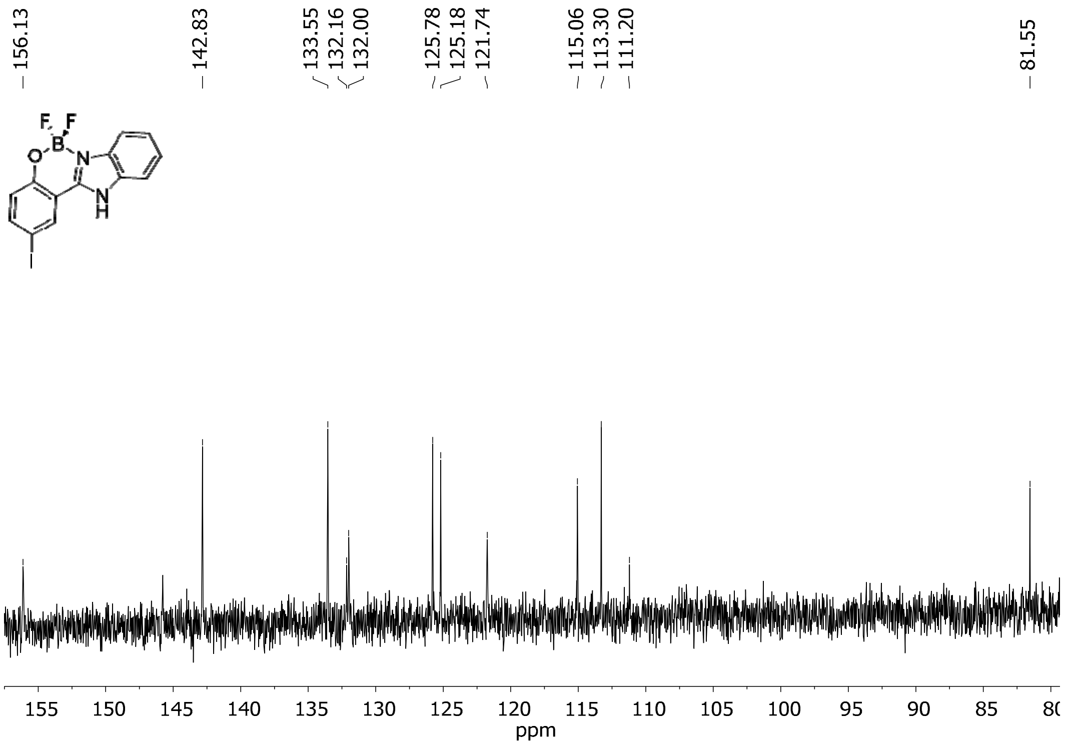

Figure S30 - ^{13}C NMR spectrum expansion of 2-iodo-6,6-difluoro-6,12-dihydrobenzo[*e*]benzo[4,5]imidazo[1,2-*c*][1,3,2]oxazaborinin-7-ium-6-uide **8d** in DMSO-d_6.

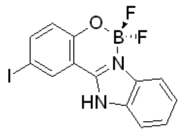

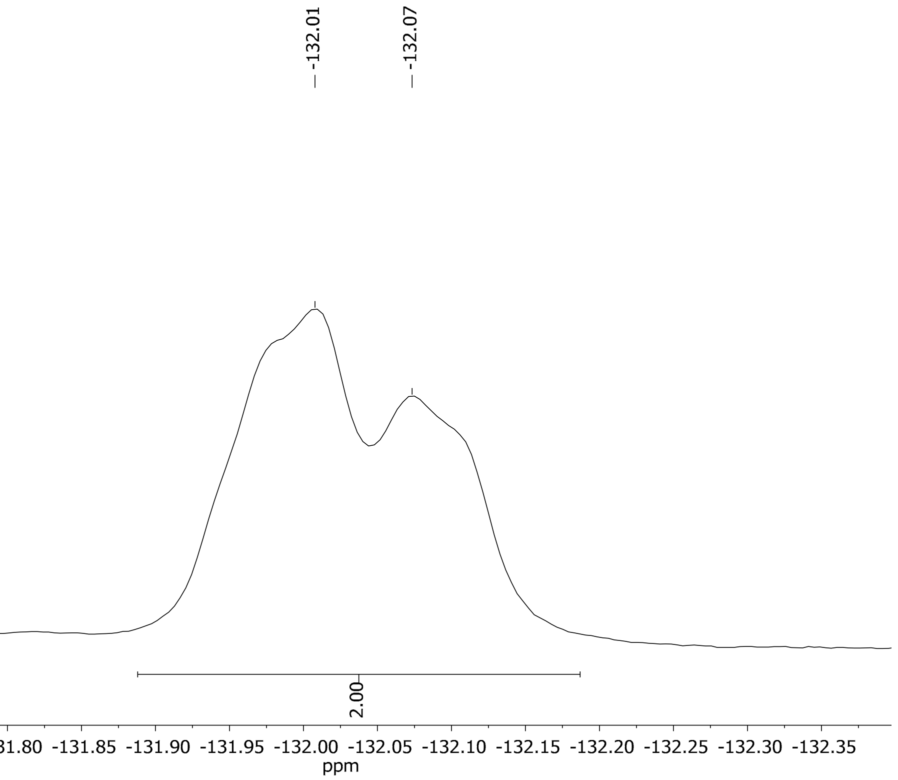

Figure S31 - ^{19}F NMR spectrum expansion of 2-iodo-6,6-difluoro-6,12-dihydrobenzo[*e*]benzo[4,5]imidazo[1,2-*c*][1,3,2]oxazaborinin-7-ium-6-uide **38d** in DMSO-d_6.

Absorption, emission and excitation spectra in solution

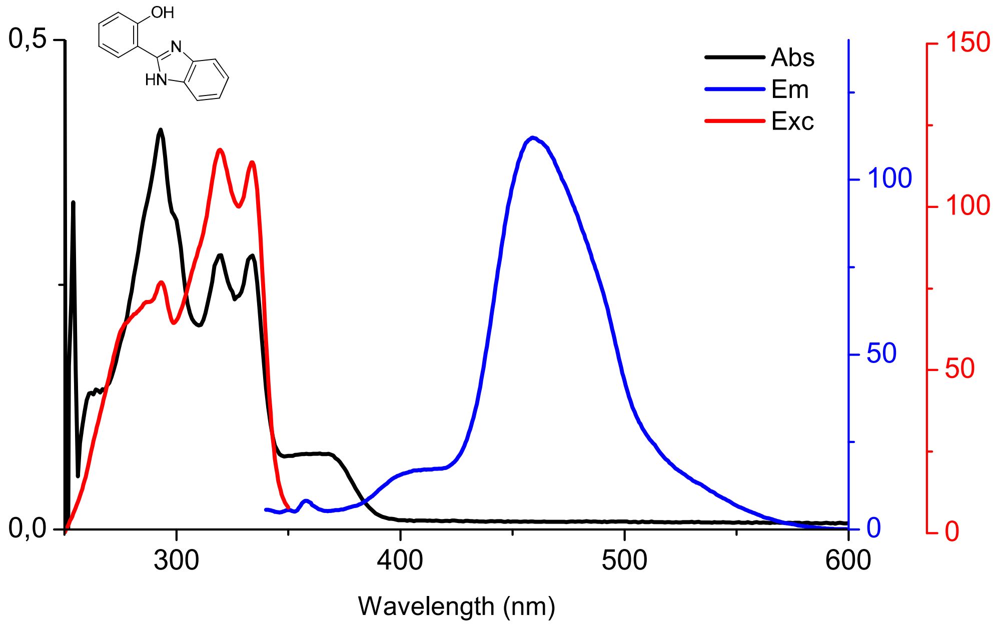

Figure S32- Absorption (black line), excitation (red line) and emission (blue line) spectra of **7a** at room temperature (c.a 5x10^{-5}M in DMSO).

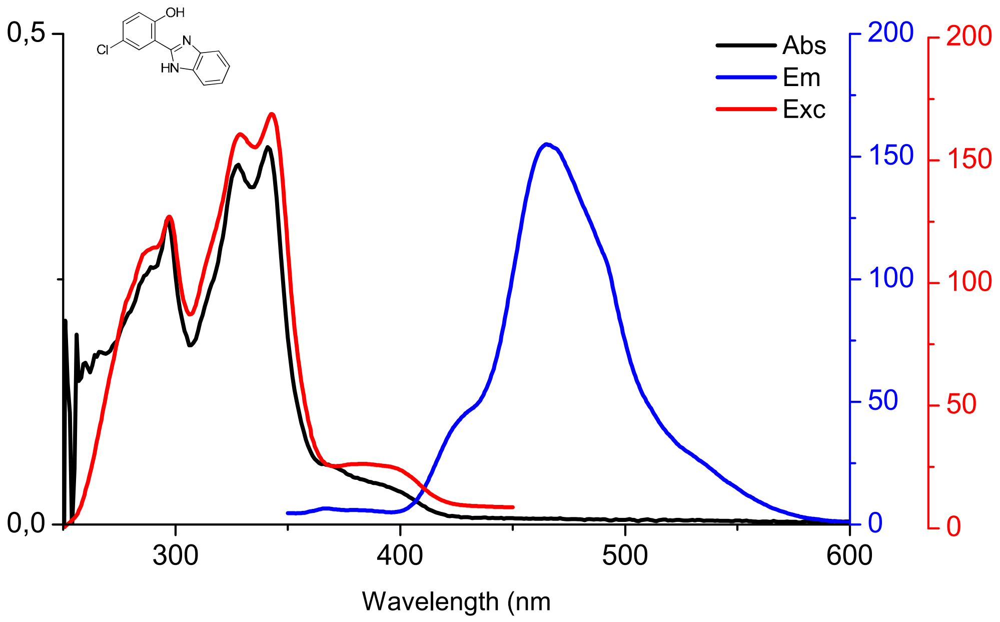

Figure S33- Absorption (black line), excitation (red line) and emission (blue line) spectra of **7b** at room temperature (c.a 5x10^{-5}M in DMSO).

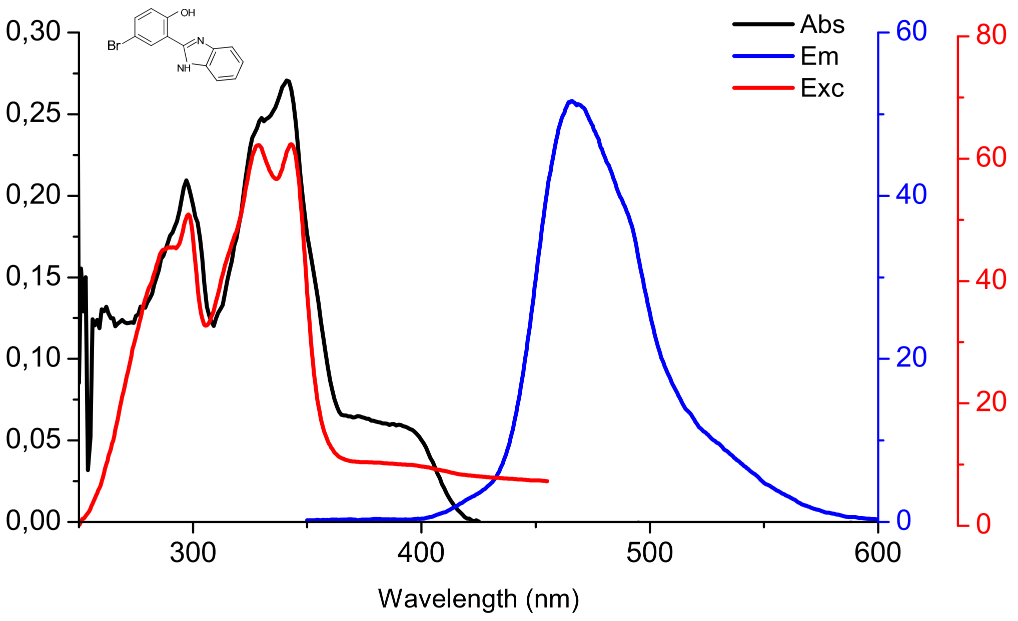

Figure S34 - Absorption (black line), excitation (red line) and emission (blue line) spectra of **7c** at room temperature (c.a 5x10^{-5}M in DMSO).

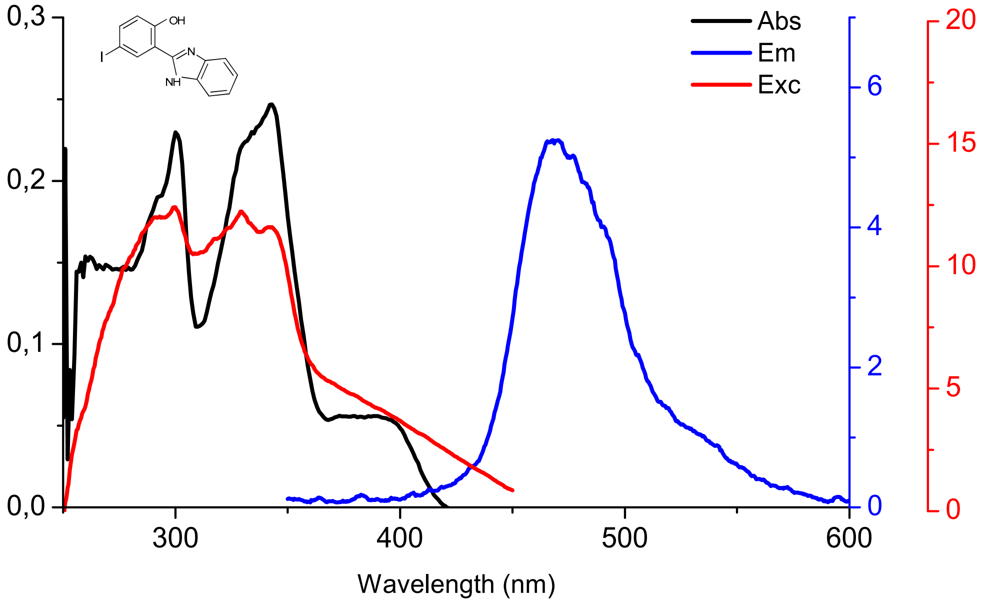

Figure S35- Absorption (black line), excitation (red line) and emission (blue line) spectra of **7d** at room temperature (c.a 5x10^{-5}M in DMSO).

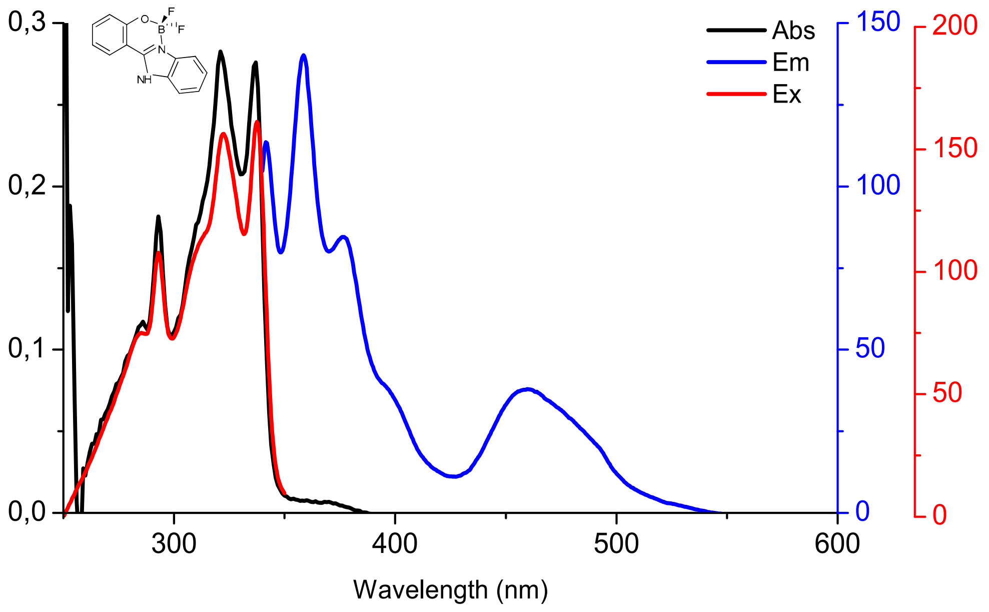

Figure S36- Absorption (black line), excitation (red line) and emission (blue line) spectra of **8a** at room temperature (c.a 5x10^{-5}M in DMSO).

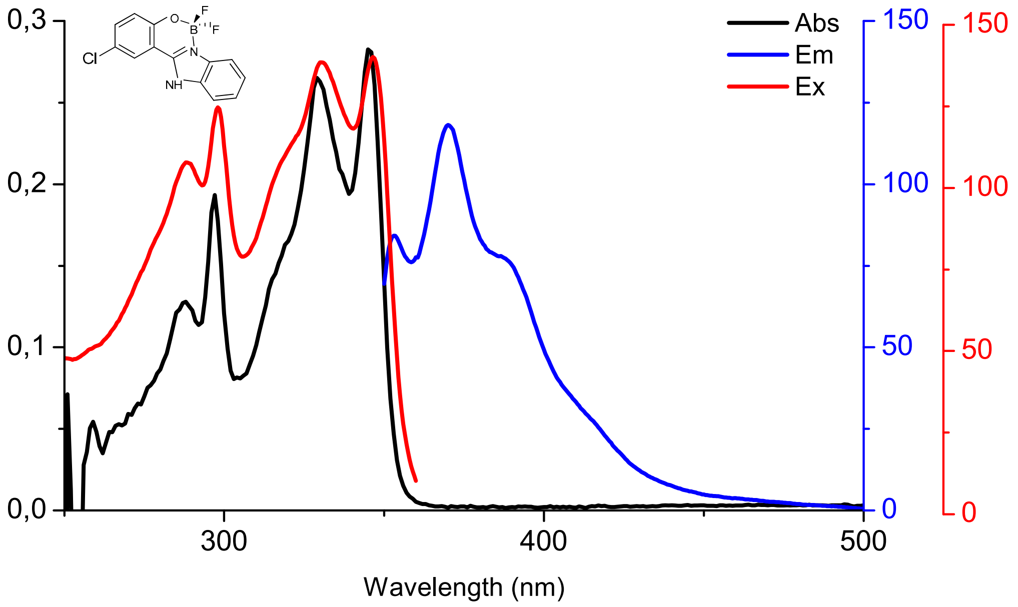

Figure S37- Absorption (black line), excitation (red line) and emission (blue line) spectra of **8b** at room temperature (c.a 5x10^{-5}M in DMSO).

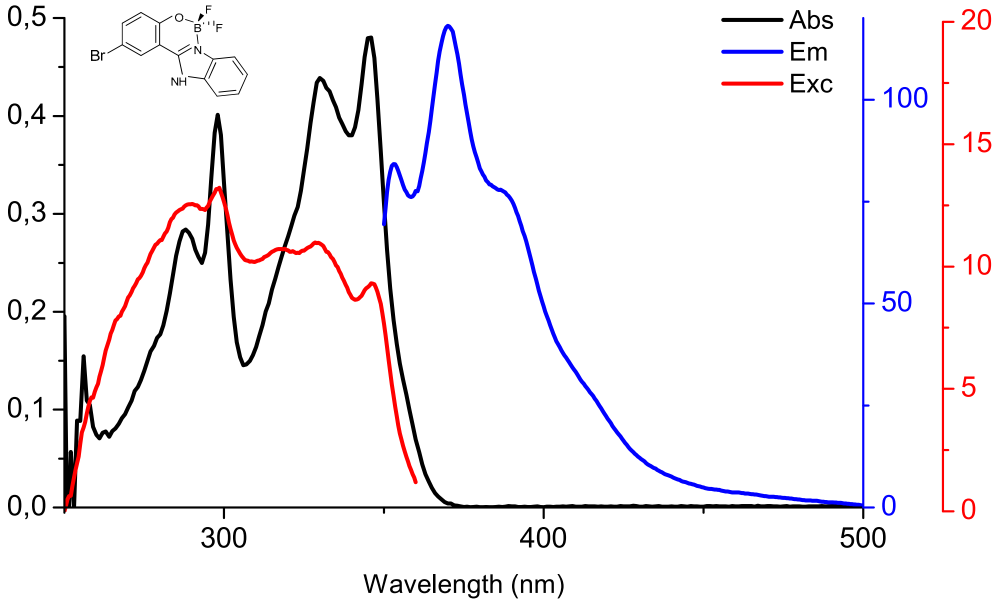

Figure S38- Absorption (black line), excitation (red line) and emission (blue line) spectra of **8c** at room temperature (c.a 5x10^{-5}M in DMSO).

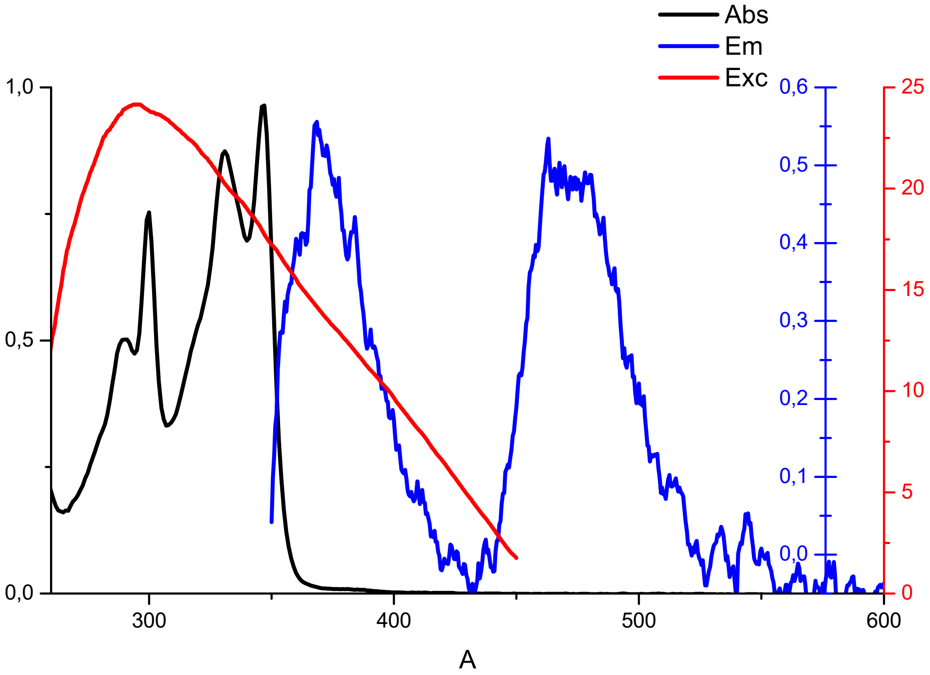

Figure S39 - Absorption (black line), excitation (red line) and emission (blue line) spectra of **8d** at room temperature (c.a 5x10^{-5}M in DMSO).

Excitation and emission spectra in solid state

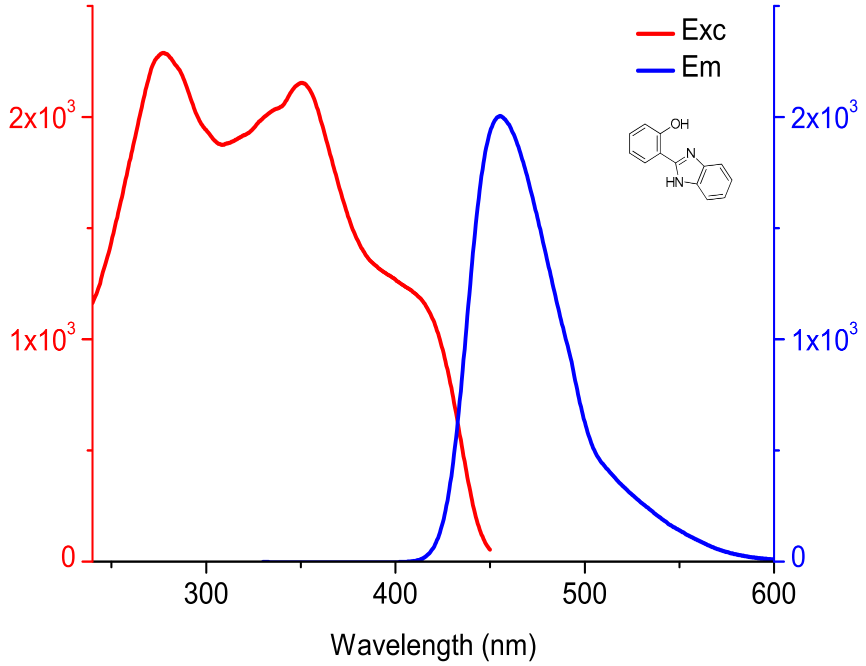

Figure S40 - Excitation (red line) and emission (blue line) spectra of **7a** at room temperature in solid state.

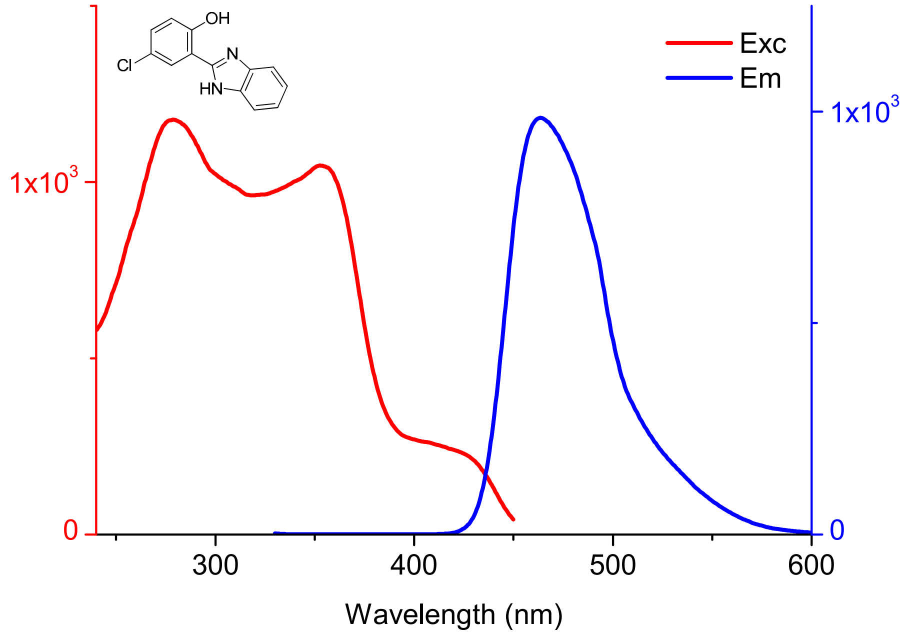

Figure S41 - Excitation (red line) and emission (blue line) spectra of **7b** at room temperature in solid state.

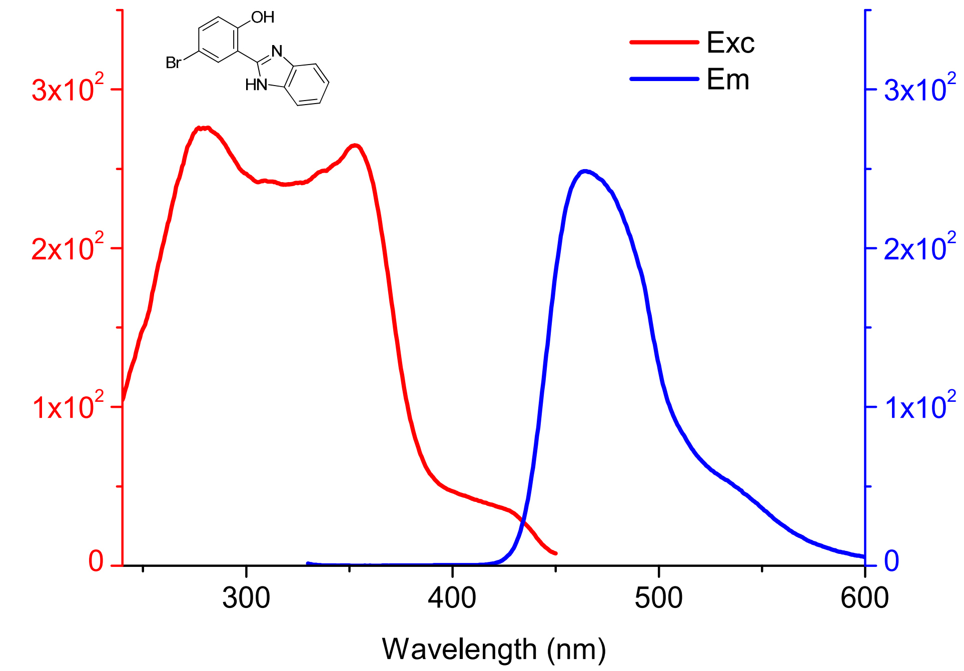

Figure S42- Excitation (red line) and emission (blue line) spectra of **7c** at room temperature in solid state.

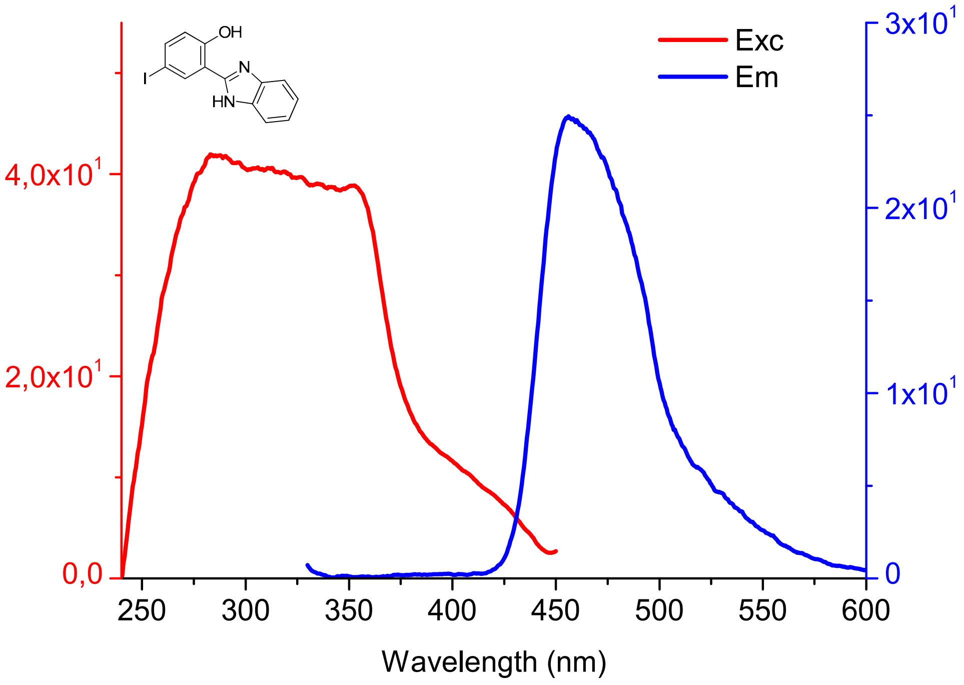

Figure S43- Excitation (red line) and emission (blue line) spectra of **7d** at room temperature in solid state.

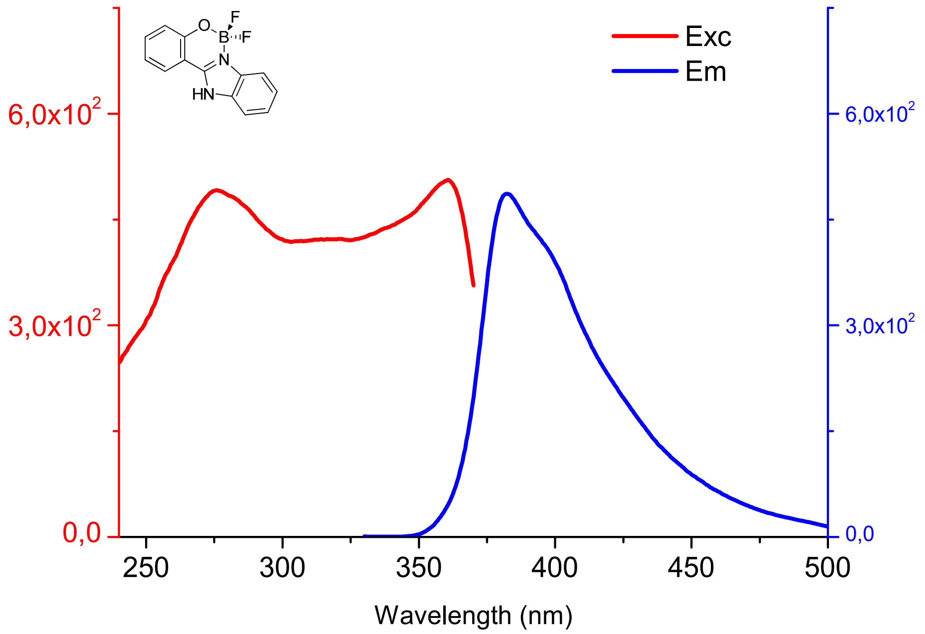

Figure S44- Excitation (red line) and emission (blue line) spectra of **8a** at room temperature in solid state.

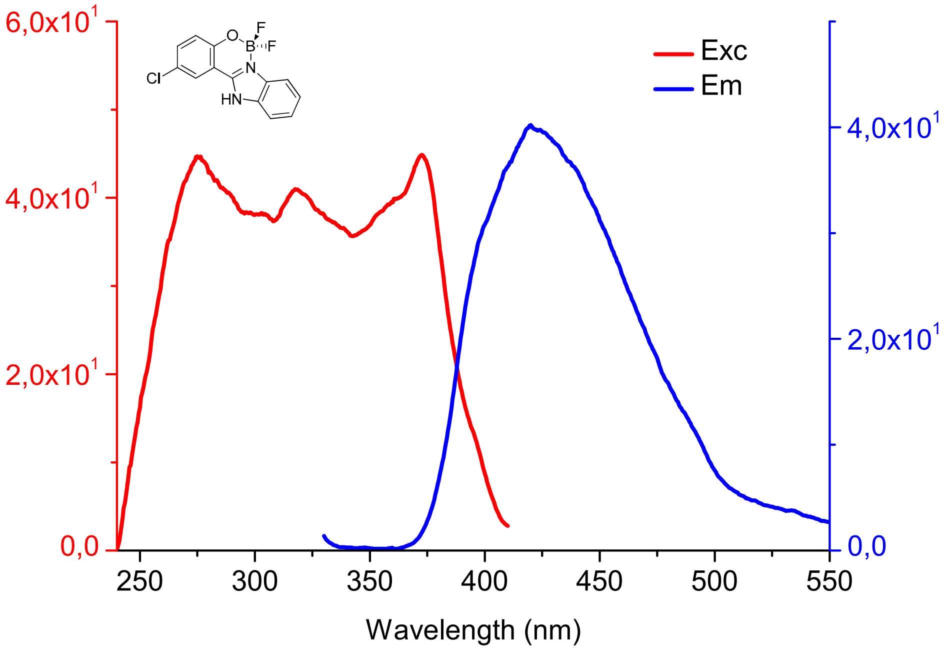

Figure S45- Excitation (red line) and emission (blue line) spectra of **8b** at room temperature in solid state.

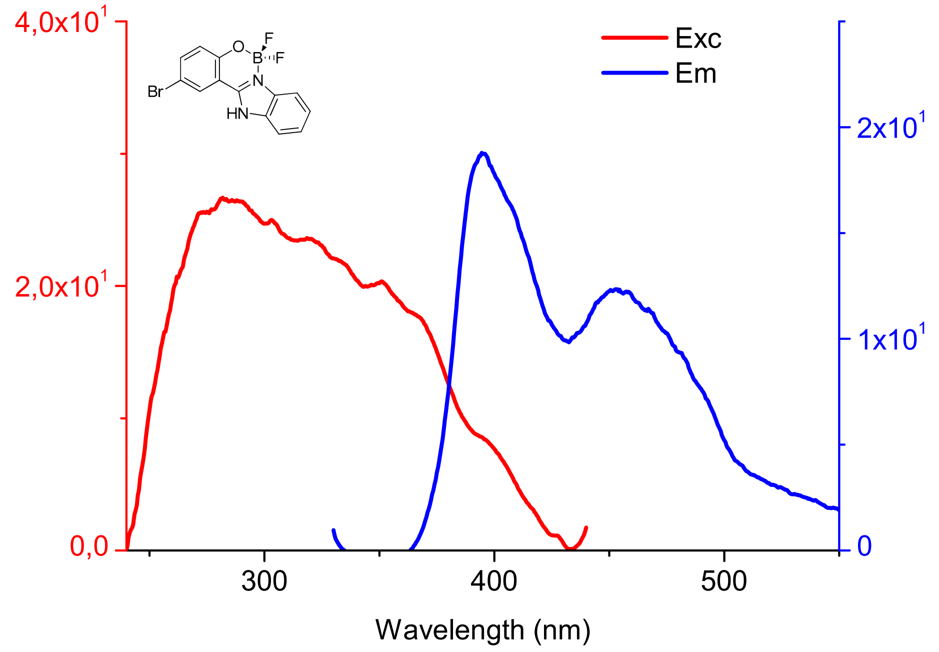

Figure S46 - Excitation (red line) and emission (blue line) spectra of **8c** at room temperature in solid state.

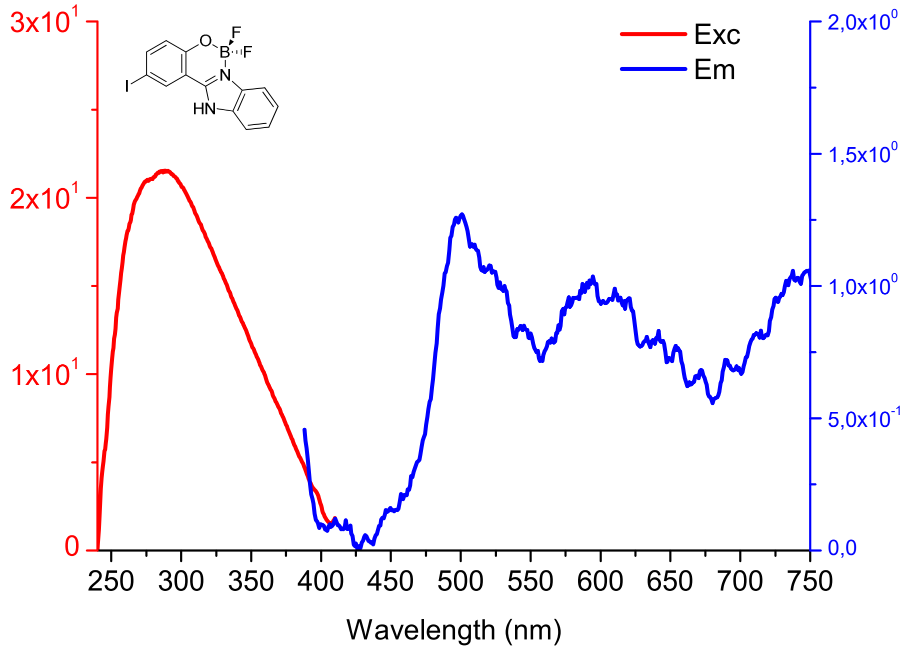

Figure S47 - Excitation (red line) and emission (blue line) spectra of **8d** at room temperature in solid state.
